# Combining Evidence of Preferential Gene-Tissue Relationships from Multiple Sources

**DOI:** 10.1371/journal.pone.0070568

**Published:** 2013-08-12

**Authors:** Jing Guo, Mårten Hammar, Lisa Öberg, Shanmukha S. Padmanabhuni, Marcus Bjäreland, Daniel Dalevi

**Affiliations:** 1 Department of Medical Biochemistry and Biophysics, Karolinska Institute, Stockholm, Sweden; 2 Cardiovascular & Gastrointestinal iMed, AstraZeneca R&D Mölndal, Mölndal, Sweden; 3 Respiratory, Inflammation & Autoimmune iMed, AstraZeneca R&D Mölndal, Mölndal, Sweden; 4 Digital Enterprise Research Institute, IDA Business Park, Galway, Ireland; 5 R&D Information, AstraZeneca R&D Mölndal, Mölndal, Sweden; 6 Biometrics and Information Sciences, AstraZeneca R&D Mölndal, Mölndal, Sweden; University of Lausanne, Switzerland

## Abstract

An important challenge in drug discovery and disease prognosis is to predict genes that are preferentially expressed in one or a few tissues, i.e. showing a considerably higher expression in one tissue(s) compared to the others. Although several data sources and methods have been published explicitly for this purpose, they often disagree and it is not evident how to retrieve these genes and how to distinguish true biological findings from those that are due to choice-of-method and/or experimental settings. In this work we have developed a computational approach that combines results from multiple methods and datasets with the aim to eliminate method/study-specific biases and to improve the predictability of preferentially expressed human genes. A rule-based score is used to merge and assign support to the results. Five sets of genes with known tissue specificity were used for parameter pruning and cross-validation. In total we identify 3434 tissue-specific genes. We compare the genes of highest scores with the public databases: PaGenBase (microarray), TiGER (EST) and HPA (protein expression data). The results have 85% overlap to PaGenBase, 71% to TiGER and only 28% to HPA. 99% of our predictions have support from at least one of these databases. Our approach also performs better than any of the databases on identifying drug targets and biomarkers with known tissue-specificity.

## Introduction

In pharmaceutical development the understanding of gene expression across human tissues is highly relevant in numerous stages. For example,

- In Target Selection, to ensure that the potential drug target is expressed only in the relevant tissues.
- In Biomarker identification, to ensure that measurable analytes originate from the right source tissue.
- To ensure that drug transporters and drug metabolic proteins are expressed in relevant tissues.

It is well known that finding suitable targets, biomarkers and proteins involved in drug transport and metabolism impacts the success rate of late stage clinical trials.

Historically, most drugs have been designed to target proteins without any detailed and extensive knowledge about the protein's global expression within the body. Only a few drugs in clinical use target proteins encoded by genes preferentially expressed in only one tissue, e.g. omeprazole (ATP4A), flecainide (SCN5A), orlistat (PNLIP, LIPF), methimazole (TPO) and dapagliflozin (SLC5A2) - for more information regarding these see DrugBank [1]. In recent years the interest has grown for biomarkers for the purpose of prognosis, monitoring of disease progression, effect of treatment, or for patient stratification. Since biomarkers for practical reasons are measured in blood plasma or serum, the specific source of the analyte is crucial (besides being responsive to disease activity). One of the most commonly used biomarkers in the clinic since long time back [2], [3] is c-reactive protein encoded by CRP, which is uniquely expressed in the liver in response to inflammatory processes or tissue damage in the body. Other examples of tissue-specific and more disease-specific blood biomarkers are prostate-specific antigen (PSA), encoded by the KLK3 gene uniquely expressed in the prostate, and cardiac troponin T expressed from the TNNT2 gene in the heart.

A growing number of techniques allowing for extensive, detailed, and sensitive quantification of gene expression gives the opportunity to identify genes that are preferentially expressed in a single tissue so called *tissue-specific* genes or *tissue-selective genes* that are constrained to a limited number of similar tissues [4]. This is an important auxiliary step in the process of developing new drugs and biomarkers. Although several public resources exist for predicting whether genes are tissue specifically expressed, the knowledge remains low and one cannot rely on an individual data source or database to find reliable answers.

Data obtained from different experiments will vary depending on the applied technology, the experimental design and the biological context. The combination of multiple datasets enables assessing hypotheses in a context-independent setting where study-specific biases are undermined. Basically two different approaches can be taken where data is either combined at the data-level (low-level), where raw data sets are integrated, or at the interpretative level (high-level), where the outcome from several independent analysis are combined [5]. Microarray analysis is a well established technology where the amount of data is growing rapidly. Different groups are often conducting experiments aiming at resolving the same or similar scientific questions but uses different protocols, platforms, data formats and analysis methods. Both high- and low-level integration are used in practice [6], [7], [8], [9], [10], [11], [12], [13] and there they have different advantages and limitations. High-level integration has the important advantage that it allows for mixing data obtained from different technologies, e.g. data from microarray and RNA-seq.

In this study we combine datasets from multiple sources at the high-level to discriminate between genes that are *specific*, *2-selective* (preferentially expressed in two tissues) and those that are *ubiquitously expressed* (i.e. more than two tissues). We use four human microarray datasets together with three different methods. The methods are trained and tested on positive and negative gene sets using cross-validation. The output from each method is combined using a consensus vote. Finally, we run the selected methods on the entire datasets and output a combined list of results using a rule-based score.

## Materials and Methods

### Datasets

Four human microarray datasets were used: GNF1H (Human U133A/GNF1H Gene Atlas) [14], NCBI GEO GDS3113 [15], NCBI GEO GSE7307 [16] and GeAZr (licensed from GeneLogic). The CEL files from GEO were downloaded and processed in R (version 2.9) using Bioconductor (version 2.4). Only normal human tissue samples were selected for the analysis. All datasets except GDS3113 have been processed by the MAS5 algorithm and log_2_ transformed before further analyses. GDS3113 were normalized using the Limma method (see [15] for details). Table 1 lists some additional information regarding the datasets.

**Table 1:** Some additional information regarding to the 5 datasets.

DATA SOURCE	NUMBER OF PROBE SETS	NUMBER OF TISSUES BEFORE MAPPING	NUMBER OF TISSUES AFTER MAPPING	DATA TYPE
**GNF1H**	22283	84	34	HG-U133a
**GDS3113**	44928	32	27	ABI Human Genome Survey Microarray
**GeAZr**	32878	100	55	HG-U133a,b
**GSE7307**	53998	105	42	HG-U133plus2

### ROKU-SPM

ROKU [17] uses Shannon entropy to predict whether a gene is specifically or ubiquitously expressed across a set of tissues. If a gene is predicted specific, an outlier detection method is used to identify the tissue [18]. Another measure, SPM, is used by the PaGenBase database [19]. It is a normalized specificity measure, based on the expression vector, which is between 0 and 1. A value close to 1 indicates specificity and a value close to 0 indicates ubiquitous expression.

We found that both ROKU and SPM had some limitations with respect to some data patterns. ROKU can easily be modified to incorporate the SPM value as a parameter to overcome these limitations. Basically both SPM and the entropy are used to predict whether a gene is specific, 2-selective or ubiquitously expressed. Two SPM values and the entropy are used as parameters in the optimization (see Text S1 for a detailed description). The modified ROKU-SPM method has been used in the analyses.

### Decision function

The decision function [20] uses the gap (*g*) between the intensities, a specificity value (*sp*), and a decision value (*d*) to determine whether a gene is preferentially expressed or not. *g*, *d* and *sp* are used as parameters in the optimization.

### Bayes factor

The Bayes factor approach [21] quantifies how much evidence there is to support specificity. This method can only be applied to datasets with multiple samples per tissue.

The hypotheses tested are:
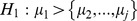
against the alternative
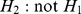
Where 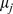 denotes the population mean of tissue *j*. Note, 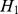 corresponds to that the gene is tissue-specific. We have updated the method to also test the hypothesis of 2-selectivity where we use the following null-hypothesis
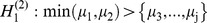
Further, we found that the variance between the replicates of each tissue is very small compared to the variability between the tissues, which resulted in support for 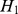 for almost all genes. The variability of the replicates gives a good estimate of the reproducibility of the experiment but it neglects the magnitude of the differences with respect to the variance between the tissues. We therefore modified 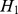 to also incorporate this magnitude by testing
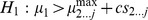
where *c* is a constant, 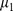 is the maximum mean over all tissues, 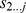 is the sample standard deviation of tissue 2 to *j* and 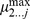 is the maximum mean over tissue 2 to *j*. This means that it is not enough to have a mean greater than the others, it also needs to exceed the others by a factor times the variability that is observed between the other tissues. The same modification was done to 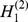,
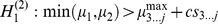
where 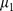 and 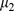 are the largest means over all tissues, 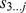 is the sample standard deviation of tissue 3 to *j* and 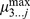 is the maximum mean over tissue 3 to *j*. We have modified the software in [21] to incorporate these changes. Simulations, as described [21], were used to find the Bayes factor thresholds 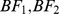 (see [21]) and the constant *c*. A detailed description of this method is available at the Chalmers online library [22].

### Optimization function

A simple score (*s*) was used for evaluating how well a method (*i*) performs on the training data:
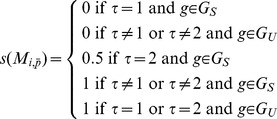
where 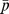 is a parameter vector and *τ* is the number of tissues identified by method *i* given 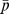 and a gene *g* from either the specific training set 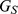 (positive set) or the ubiquitous training set 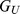 (negative set).

The function has a minimum if the method identifies one tissue for each of the genes in 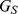 and no preferential tissues for the genes in 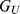. Therefore, we seek the vector 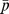 that minimizes the sum of *s* of all training genes.

### Training and test gene sets

Training and test gene sets were retrieved from the HuGEindex.org database [23]. Five disjoint datasets were selected from the “Tissue Selective Genes” section (in Text S1) which in total contains seven tissues. The genes were picked in a random fashion and the expression patterns were manually investigated to exclude genes which had an expression pattern that contained no signal for specificity, i.e. not visually detectable by a manual curator or any of the methods with default parameters. The datasets corresponded to different tissues in order to identify/verify robust parameter settings: 1) Mixed tissues, 2) Kidney specific, 3) Muscle specific, 4) Lung or prostate specific and 5) Liver specific. In each dataset we also added 10 ubiquitously expressed genes from the “House Keeping Genes” section, also from HuGEindex.org. The five training/test gene sets are available in the supplementary materials (Table S2, S3, S4, S5, S6). The gene set containing mixed tissues is shown in Table 2 where the first 20 genes are specific (i.e. belong to 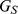) and the last 10 are ubiquitous (i.e. belong to 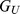). All datasets were used both for training and testing. Parameters were estimated from one datasets and were then used to predict genes in the other datasets (testing).

**Table 2:** Predicted tissues after optimization on the mixed set of training genes.

GENE	GNF1H	GeAZr	GSE7307	GDS3113	TISSUE ANNONTATION
**FXYD2**	**T**	**T**			T = Kidney
**PAX8**	**T**	**T**		**T**	T = Thyroid
**HABP2**	**T**	**T**	**T**	**T**	T = Liver
**SAA4**	**T**	**T**	**T**	**T**	T = Liver
**CPN2**	**T**	**T**	**T**	**T**	T = Liver
**ASGRT**	**T**	**T**	**T**	**T**	T = Liver
**LIPC**	**T**	**T**	**T**		T = Liver
**SFTPC**	**T**	**T**	**T**	**T**	T = Lung
**SFTPB**	**T**	**T**	**T**	**T**	T = Lung
**KLK2**	**T**	**T**	**T**	**T,S**	T = Prostate, S = Salivary gland
**ACPP**	**T**	**T**	**T**	**T**	T = Prostate
**CA3**	**T**	**T**	**T**	**T**	T = Thyroid
**APOC3**	**T**	**T**	**T,S**	**T,S**	T = Liver, S = Small intestine
**KLK3**	**T**	**T**	**T**	**T**	T = Prostate
**LOR**	**T**	**T**	**T,S**	**T**	T = Skin, S = Vulva
**ENO3**	**T**	**T**	**T**	**-**	T = Heart, S = Muscle
**ITIH2**	**T**	**T**	**T**	**T,S**	T = Liver, S = Spinal cord
**DPEPT**	**T,S**				T = Kidney, S = Pancreas
**TTR**		**T**	**T,S**		T = Liver, S = Retina
**USPT3**	**T**	**T**		**T**	T = Muscle
**VIM**					
**RPL4T**					
**E2F4**					
**XPOT**					
**UQCRH**					
**SEPWT**					
**YWHAQ**					
**PSD**					
**PSMB5**					
**CFLT**					

T and S represent predicted tissues per gene after consensus voting (See method section). Empty white means one of the following: ubiquitously expressed, no tissue with strong support or no data. Bold indicates that the predicted result is exactly (T), or partial agreement (T,S), the same as the HuGEindex.org database. A more detailed table is shown in the supplementary material where results from individual methods are presented (Table S2).

### Training schema

We used the set of parameters, given in the previous sections, for each method in a simple optimization schema (see Text S1). The set of parameters that minimized the optimization function was chosen.

### Vocabulary mapping

The four data sources use their own tissue vocabularies. GNF1H, GeAZr, GDS3113 and GSE7307 with 84, 100, 32 and 105 tissue-terms respectively. Although many of the tissues are shared in all datasets, it is necessary to reorganize the terms to enable further analyses of the data. In our analyses we remove tissues that are out of scope and group tissues that are functionally or literary similar. We used the Brenda vocabulary [24] and the four datasets were mapped onto this vocabulary. Some tissues, such as adrenal gland and adrenal cortex, were grouped together and the max intensity was used to represent the grouped tissues (see Table S1 for more details about the groups).

### Consensus vote for each dataset

Two or three methods were applied on each dataset depending on whether there were multiple samples per tissue – a requirement for the Bayes factor method. A consensus vote was obtained by combining the result from all methods, using the rules described in Table 3, to determine if a gene is specific, 2-selective or ubiquitous.

**Table 3:** Rules for the consensus vote.

Method 1	Method 2	Method 3	Consensus vote
T	T	T	**T**
T	T	T,S	**T**
T	T	Uq	**T**
T	T		**T**
T	T,S	T,S	**T,S**
T	T,S	Uq	**T**
T	T,S		**T**
T	Uq	Uq	**Uq**
T	Uq		**T**
T,S	T,S	T,S	**T,S**
T,S	T,S		**T,S**
T,S	T,S	Uq	**T,S**
T,S	Uq	Uq	**Uq**
T,S	Uq		**T,S**
Uq	Uq	Uq	**Uq**
Uq	Uq		**Uq**

The results from two or three different methods are combined to determine if a gene is specific (T), 2-selective (T,S) or ubiquitous (Uq). T and S are the identified tissues.

### Combining the output from several datasets

A gene is often represented by several probe sets that may be conflicting with each other and there is no universal rule telling us which of them to trust more. We have, however, designed a score that will capture some of the evidence researchers look for when analyzing several probe sets. For example, if three out of five probe sets indicate that a gene is specifically expressed in the liver and the two remaining indicate ubiquitously expressed, we believe there is enough evidence that points towards specificity for liver. These rules will be captured by the *inner-score*, 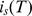, for tissue *T* and gene *s*. 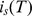 is calculated for each gene by the following steps:

1. If at least 50% of the probe sets are either specific or 2-selective and contains the same tissue T, remove all probe sets indicating ubiquitously expressed – we regard them as *non-informative*.
2. If at least 50% of the remaining probe sets are either specific for the same tissue (*T*) or 2-selective for the same two tissues 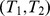, then 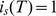, for that tissue, or 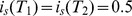 for the two tissues. Otherwise, 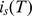, for each detected tissue, will be the average of the frequency of the tissue over the probe sets.

**Figure 1:**
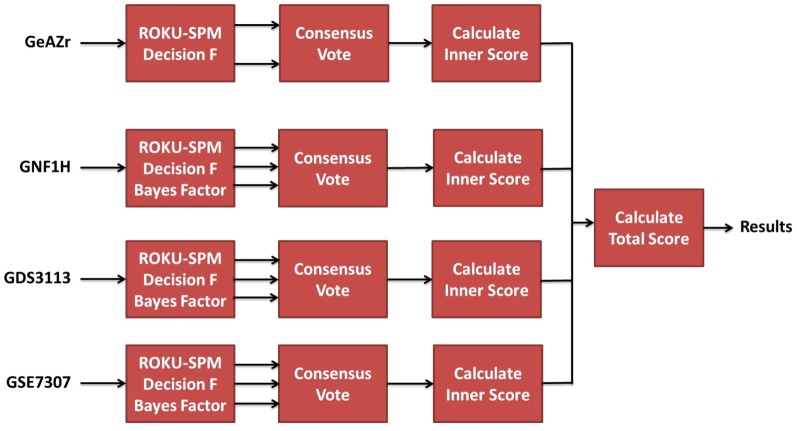
Flow diagram illustrating the procedure for predicting preferentially expressed genes from multiple datasets. The following steps are taken: 1) The methods (Bayes Factor, ROKU-SPM and Decision F = Decision function) are applied to each dataset separately, 2) The consensus vote combines the output from the methods, 3) The inner-score combines the output from several probe sets and 4) The total score integrates the results from all datasets into a common result.

The *total-score*, 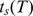, for a gene *s* and tissue *T*, will be the average over the inner-scores, hence constrained between 0 and 1, over all datasets. A flow diagram summarizing the whole procedure is shown in Figure 1.

Two examples on how to calculate the score are shown below for illustrative purposes.

#### Example 1: GRHPR

**Figure 2:**
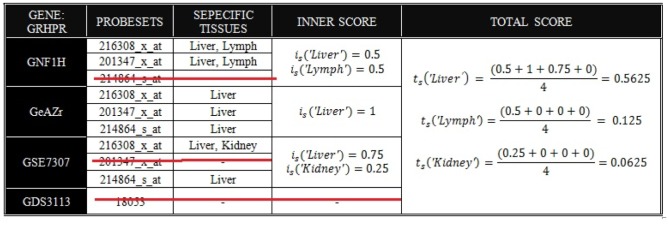
Example GRHPR of how the total score is calculated (see main text).

There are three probe sets representing the gene GRHPR in GNF1H, GeAZr and GSE7307, and one probe set in GDS3113. First, the inner score 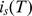 is calculated for each data source separately. In GNF1H, two probe sets out of three detect the gene as specific in Liver and Lymph, so the probe set 214864_s_at (marked with the red line, Figure 2) is regarded as non-informative (Rule 1), and at least 50% of the remaining probe sets indicate Liver and Lymph selective, so the inner score will be 0.5 for Liver and 0.5 for Lymph (Rule 2). In GeAZr, as the three probe sets are all detected as Liver specific, the score for Liver will be 1. In GSE7307, both 214864_s_at and 216308_x_at are detected as Liver specific/2-selective, thus probeset 201347_x_at (marked with the red line in Figure 2) will be regarded as non-informative and the score for Liver and Kidney will be the average of 214864_s_at and 216308_x_at (Rule 2). For GDS3113, this gene is ubiquitously expressed. Second, the total score 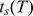 of a tissue is the average of the all inner scores (sum of scores for each tissue divided by 4).

#### Example 2: ASS1

**Figure 3:**
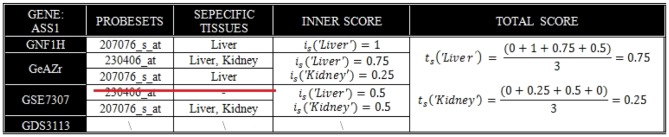
Example ASS1 of how the total score is calculated (see main text).

The gene ASS1 is represented by one probe set in GNF1H and by two probe sets in GeAZr and GSE7307, but none in GDS3113. The inner scores in each data sources are shown in Figure 3. In GeAZr scores for Liver and Kidney are averaged from the two probe sets. In GSE7307, one probe set out of two indicates that the gene is 2-selective (Liver and Kidney) so the ubiquitously expressed probe set (230406_at, marked with the red line in Figure 3) is therefore non-informative (Rule 1) and will be disregarded from further calculations. Finally, the total score (per tissue) is obtained by averaging over the inner scores over the four datasets.

### Public databases

We compare some of our results with the publically available databases: PaGenBase, TiGER and HPA. In PaGenBase we use 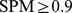 as cutoff (the default value). In TiGER we set the enrichment score to be greater than 5 and the P-value to be less than 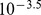. In HPA we interpret “Strong” (Level of antibody staining) or “High” (level of annotated protein expression) as indicators of expression in a given tissue. HPA is continuously updating while PaGenBase and TiGER are static.

### Software

R and Perl scripts to perform the analyses in this study are provided with instructions at GIThub: https://github.com/ddalevi/tissue-specificity

## Results

### Training and optimization

We used five distinct training sets, each having a set of specifically and ubiquitously expressed genes, with respect to different tissues: 1) Mixed tissues, 2) Kidney specific, 3) Muscle specific, 4) Lung or prostate specific and 5) Liver specific. Ideally, the methods should output a single tissue; in some cases also two tissues, but all other number of outputs should be penalized by the optimality criterion (see Optimization function in Methods). Parameters of all method/dataset combinations were estimated from each of the training gene sets separately resulting in five different sets of parameters. The training results from the genes of mixed tissues are shown in Table 2 (the others are shown in the supplementary materials Table S3, S4, S5, S6). Each of the five sets of parameters was evaluated (tested) on the four datasets not used for training (see Table 4). For example, when training on the Liver dataset we obtained a set of optimal parameters that resulted in 95% correctly predicted genes in the liver dataset. When this set of parameters was applied to the other datasets (for testing) we obtained 95% (Mixed), 95% (Kidney), 95% (Muscle) and 94% (Lung or prostate). This all-against-all procedure was done to verify the robustness of the parameters, i.e. what impact will a slight change in parameter values have on the predictions. As seen in Table 4, we only observed minor differences between the sets of parameters and it will be hard to argue that one set is better than another. Therefore, we decided to use the sets of parameters obtained from the mixed tissue set as this represents the most general setting. The complete results from each of the datasets together with outputs from individual methods are presented in the supplementary material (Table S2). Note that the Bayes factor method can only be applied to some of the datasets where we have multiple samples per tissue. Most methods performed quite well and in many cases all methods could identify the correct tissue given by HuGEindex.org (T in Table S2, S3, S4, S5, S6). The consensus vote (Table 2) resulted in better predictions than those obtained from the individual methods.

**Table 4:** Training/Testing.

Training parameter\Testing dataset	Mixed tissues	Kidney	Muscle	Lung & Prostate	Liver
**Mixed tissues**	91%	95%	95%	83%	95%
**Kidney**	90%	95%	93%	83%	95%
**Muscle**	91%	93%	95%	82%	95%
**Lung & Prostate**	90%	94%	93%	83%	94%
**Liver**	91%	94%	93%	81%	95%

The percentage shows the agreement of the detection and HuGEindex prediction. Specific and ubiquitously expressed genes are all considered as positive if agrees with the prediction. The rows show the dataset used for training, i.e. to estimate the parameters. The columns show the testing on the other datasets.

**Figure 4:**
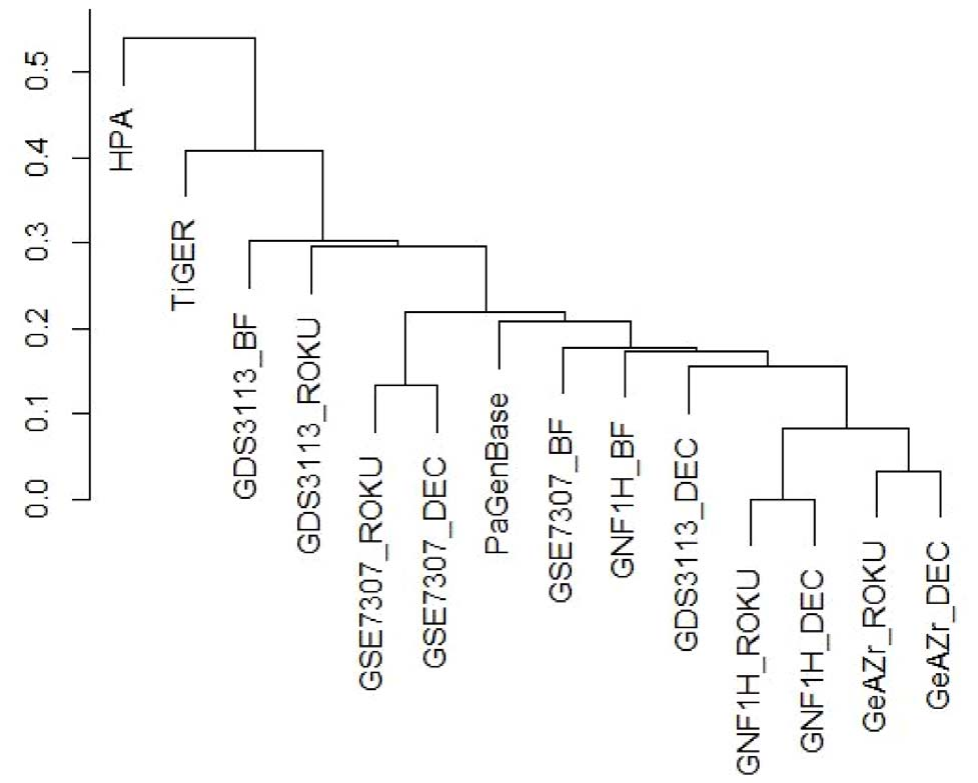
Clustering of data based on the 30 training genes using the optimal parameters.

We also clustered the data, based on the training set, to illustrate that the results cluster based on dataset rather than method (Figure 4), which stresses the fact that data is more important than choice of method, i.e. given optimal parameters. We also added HPA, TiGER and PaGenBase data in the plot. PaGenBase, which is based on microarray data, is closer to our results than HPA and TiGER (protein and EST data).

### Combined output of all genes

The optimal parameters for the five datasets were used to apply the methods on the full datasets. The total-score 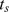 was used to combine the datasets for each gene. The output genes were divided into separate groups based on the score. The specific genes ended up in four groups: 1) 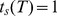; *strong support*, 2) 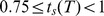; *high support*, 3) 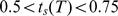; *medium-high support* and 4) 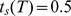; *medium support*. The 2-selective genes were divided into two groups: 1) 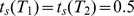; *strong support* and 2) 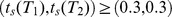; *medium-high support.* Further, we also categorize the genes based on how many datasets that contributed to the output (so called the *coverage*, Table 5). For example, *3 out of 4* in the table means that three out of four datasets were used when calculating the score (the more the better). In the previous examples of GRHPR and ASS1 (Figure 2 and Figure 3), GRHPR is predicted Liver-specific with medium-high support, i.e. 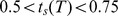, and coverage 4 out of 4. ASS1 is predicted 2-selective for Liver and Kidney with medium-strong support, i.e. 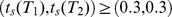, and coverage 3 out of 4.

**Table 5:** Summary of results.

Specificity	Specific				2-Selective		Total specific or 2-selective	Total
Score	Strong	High	Medium high	Medium	Strong	Medium high		
**4 out of 4**	191	306	143	498	4	27	1169	9354
**3 out of 4**	113	37	200	62	6	177	595	5179
**2 out of 4**	147	41	16	663	44	4	915	3550
**1 out of 4**	1011	6	0	0	160	2	1179	8745
**Summation**	**1462**	**390**	**359**	**1223**	**214**	**210**	**3858**	**26828**

The number of identified specific and 2-selective genes. The specific genes are divided into: strong support (score = 1), high support (0.75≤score<1), medium-high (0.5<score<0.75) and medium support (score = 0.5). The 2-selective genes are divided into: strong support (score = 0.5) and medium-high support (0.3≤score<0.5). The results are also categorized using the coverage: 4 out of 4 means that the gene exists in all four datasets, 3 out of 4 means that the gene exists in three datasets, etc. Total indicates the number of genes in each category of coverage (e.g. 9354 genes are found in all four datasets).

**Figure 5:**
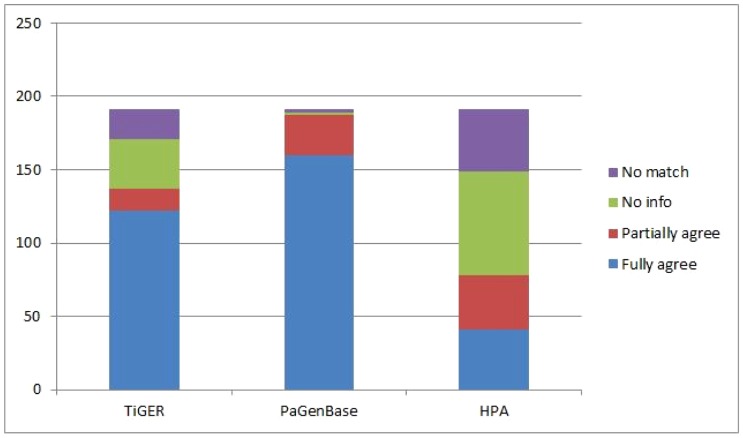
The agreement of the 191 specific genes with strong support and highest coverage, based on our predictions, to TiGER, PaGenBase (TiSGeD) and HPA. No info means missing data.

In total we found 3434 specific genes and 424 2-selective genes. It should be noted that the criteria: strong support and highest coverage used for selection of the 191 genes are very strict. We have analyzed the predicted tissues of these genes and compared them to TiGER, PaGenBase and HPA (Table 6). The overlap is, as expected, highest with PaGenBase which is a database containing tissue-specific information based on microarray data. The second highest overlap is with TiGER – the EST data – and the most dissimilar is HPA – the protein data. Our predictions in Table 6 more often agree with the consensus compared to the individual databases. For example, TPO is not predicted by TiGER but by the others, and AKAP4 is not predicted by HPA but the others, and GC is not predicted specific by PaGenBase but the others. HPA suffers a lot from missing expression data. It is clear that HPA for many genes, with well documented tissue-specificity, are output as either 2-selective or ubiquitously expressed. The agreement with our predictions is: 85% with PaGenBase, 71% with TiGER and only 28% with HPA (Figure 5). If we include the cases in HPA where the gene is strongly expressed not only in the specific tissue, but also in other tissues (partial agreement), the number is 53%.

**Table 6:** Comparison of results.

Gene	Pr	Ti	PG	HP	T	Gene	Pr	Ti	PG	HP	T	Gene	Pr	Ti	PG	HP	T
**CYP17A1**	**T**		**T**	**T+**	Adrenal	**C8G**	**T**	**T+**	**T**	*S+*	Liver	**SERPINA7**	**T**	**T**	**T**		Liver
**FDXR**	**T**		**T**	**T+**	Adrenal	**C9**	**T**	**T**	**T**	*S+*	Liver	**SERPINC1**	**T**	**T**	**T**	*S+*	Liver
**GSTA4**	**T**		**T**		Adrenal	**CPN2**	**T**		**T**	*S+*	Liver	**SERPIND1**	**T**	**T**	**T**		Liver
**HSD3B2**	**T**		**T+**		Adrenal	**CYP2A6**	**T**	**T**	**T**		Liver	**SERPINF2**	**T**	**T+**	**T**	**T+**	Liver
**NOV**	**T**		**T**	**T+**	Adrenal	**CYP2A7**	**T**	**T+**	**T+**	**T**	Liver	**SLC10A1**	**T**	**T**	**T**	*S+*	Liver
**LY6H**	**T**	**T**	**T**		CNS	**CYP2C8**	**T**	**T**	**T**	**T**	Liver	**SLC22A1**	**T**	**T**	**T+**	*S*	Liver
**CAMK1G**	**T**		**T**	**T+**	CNS	**CYP2C9**	**T**	**T**	**T**	**T**	Liver	**SPP2**	**T**	**T**	**T**		Liver
**HTR2A**	**T**		**T+**	**T+**	CNS	**CYP2E1**	**T**	**T**	**T**	**T**	Liver	**TDO2**	**T**	**T**	**T**	*S+*	Liver
**TNNT2**	**T**	**T**	**T**	**T**	Heart	**F12**	**T**	**T+**	**T**		Liver	**VTN**	**T**	**T**	**T**		Liver
**MYL7**	**T**	**T**	**T**	**T**	Heart	**F2**	**T**		**T**	*S+*	Liver	**SFTPC**	**T**	**T**	**T**	**T**	Lung
**MYBPC3**	**T**	**T**	**T**	**T**	Heart	**F7**	**T**		**T**	*S+*	Liver	**AGER**	**T**	**T**	**T**	**T+**	Lung
**MYL4**	**T**	**T**	**T**	**T+**	Heart	**F9**	**T**	**T**	**T**	*S+*	Liver	**SFTPB**	**T**	**T**	**T**		Lung
**NPPA**	**T**	**T**	**T**		Heart	**FGA**	**T**	**T**	**T**	*S+*	Liver	**SFTPD**	**T**	**T**	**T**	**T**	Lung
**NPPB**	**T**	**T**	**T**		Heart	**FGB**	**T**	**T**	**T+**	**T+**	Liver	**APOBEC2**	**T**		**T**	**T+**	Muscle
**SLC12A3**	**T**	**T+**	**T**	*S+*	Kidney	**FGG**	**T**	**T**	**T**		Liver	**MYOZ1**	**T**	**T**	**T+**	**T**	Muscle
**SLC34A1**	**T**	**T**	**T**	**T+**	Kidney	**GC**	**T**	**T**	**T**	**T+**	Liver	**AMPD1**	**T**	**T**	**T+**	**T+**	Muscle
**SLC22A6**	**T**	**T**	**T**		Kidney	**GCKR**	**T**		**T**		Liver	**MYF6**	**T**	**T+**	**T**		Muscle
**CUBN**	**T**	**T**	**T**		Kidney	**HABP2**	**T**	**T+**	**T**	S+	Liver	**NEB**	**T**		**T+**	**T**	Muscle
**KL**	**T**	**T**	**T**	**T+**	Kidney	**HAMP**	**T**		**T**		Liver	**RPL3L**	**T**	**T**	**T+**	*S+*	Muscle
**NPHS2**	**T**	**T**	**T**	**T**	Kidney	**HAO1**	**T**	**T**	**T**	**T**	Liver	**PSG2**	**T**	**T**	**T**		Placenta
**SLC22A2**	**T**	**T**	**T**	*S+*	Kidney	**HGFAC**	**T**		**T**	*S+*	Liver	**ADAM12**	**T**	**T**	**T+**	**T+**	Placenta
**SLC5A2**	**T**	**T+**	**T**	T+	Kidney	**HPR**	**T**	**T**	**T**	**T+**	Liver	**CYP19A1**	**T**	**T**	**T+**	**T+**	Placenta
**CYP1A2**	**T**		**T**	**T**	Liver	**HPX**	**T**	**T**	**T**		Liver	**EBI3**	**T**	**T**	**T**	*S+*	Placenta
**AGXT**	**T**	**T**	**T**	**T+**	Liver	**INHBE**	**T**		**T**	**T+**	Liver	**GCM1**	**T**	**T**	**T+**	**T**	Placenta
**AHSG**	**T**	**T**	**T**		Liver	**ITIH1**	**T**	**T**	**T**	*S+*	Liver	**HMGB3**	**T**		**T**		Placenta
**AKR1D1**	**T**	**T**	**T**		Liver	**ITIH2**	**T**	**T+**	**T**	**T+**	Liver	**HSD17B1**	**T**	**T**	**T**	**T**	Placenta
**ANG**	**T**	**T**	**T**		Liver	**ITIH3**	**T**	**T**	**T**	*S+*	Liver	**INSL4**	**T**	**T**	**T+**		Placenta
**APOB**	**T**	**T+**	**T**	**T**	Liver	**KLKB1**	**T**	**T**	**T**	**T+**	Liver	**LGALS13**	**T**	**T**	**T+**	*S+*	Placenta
**APOC2**	**T**	**T**	**T**	**T**	Liver	**LPA**	**T**		**T**		Liver	**LGALS14**	**T**	**T**	**T+**		Placenta
**APOF**	**T**		**T**		Liver	**MASP2**	**T**	**T**	**T**		Liver	**MAGEA8**	**T**	**T+**	**T**		Placenta
**APOH**	**T**	**T**	**T**	*S+*	Liver	**MAT1A**	**T**	**T**	**T**		Liver	**MAN1C1**	**T**	**T**	**T**	**T+**	Placenta
**ASGR1**	**T**	**T**	**T**	**T**	Liver	**MBL2**	**T**		**T**	**T**	Liver	**PLAC1**	**T**	**T**	**T**		Placenta
**ATF5**	**T**		**T**	*S+*	Liver	**NR1I3**	**T**	**T+**	**T**		Liver	**PSG11**	**T**	**T**	**T**	*S+*	Placenta
**BAAT**	**T**	**T**	**T**	**T+**	Liver	**PON1**	**T**	**T**	**T**	**T+**	Liver	**PSG3**	**T**	**T**	**T+**	*S+*	Placenta
**C2**	**T**		**T**		Liver	**PROC**	**T**	**T**	**T**	*S+*	Liver	**PSG4**	**T**	**T**	**T**	*S+*	Placenta
**C8A**	**T**	**T**	**T**		Liver	**SAA4**	**T**	**T**	**T**		Liver	**PSG5**	**T**	**T**	**T+**	*S+*	Placenta
**C8B**	**T**	**T**	**T**		Liver	**SERPINA10**	**T**	**T**	**T**	*S+*	Liver	**PSG6**	**T**	**T**	**T+**	*S+*	Placenta

Gene	Pr	Ti	PG	HP	T	Gene	Pr	Ti	PG	HP	T	Gene	Pr	Ti	PG	HP	T
**PSG7**	**T**	**T**	**T+**		Placenta	**CCIN**	**T**	**T**	**T**	**T+**	Testis	**ROPN1**	**T**	**T**	**T**	**T+**	Testis
**PSG9**	**T**	**T**	**T**	*S+*	Placenta	**CCNA1**	**T**	**T+**	**T**		Testis	**RPL39L**	**T**		**T**	**T+**	Testis
**RASA1**	**T**	**T**	**T**	T+	Placenta	**CCT6B**	**T**	**T**	**T**		Testis	**RUVBL2**	**T**		**T**	**T+**	Testis
**SIGLEC6**	**T**	**T**	**T**	T	Placenta	**COIL**	**T**	**T**	**T**	**T+**	Testis	**SLC25A31**	**T**	**T**	**T**	*S+*	Testis
**KLK2**	**T**	**T+**	**T+**	T	Prostate	**CSNK2A2**	**T**		**T**		Testis	**SLC6A16**	**T**	**T**	**T**		Testis
**PAK1IP1**	**T**		**T**	T+	Prostate	**CYLC2**	**T**		**T**		Testis	**SPANXC**	**T**		**T**	*S+*	Testis
**TGM4**	**T**	**T**	**T**	T	Prostate	**DDX4**	**T**	**T**	**T**	**T**	Testis	**SPATA6**	**T**		**T**	**T+**	Testis
**CST4**	**T**	**T**	**T**		Salivary gland	**DKKL1**	**T**	**T**	**T**	**T**	Testis	**SURF2**	**T**		**T**		Testis
**CST5**	**T**		**T+**		Salivary gland	**DMRT1**	**T**	**T**	**T**	*S*	Testis	**TAF7L**	**T**	**T**	**T**	**T**	Testis
**CA6**	**T**		**T+**	T	Salivary gland	**FBXO24**	**T**	**T**	**T**	**T+**	Testis	**TBL2**	**T**		**T**	**T+**	Testis
**CST2**	**T**		**T**	T	Salivary gland	**GK2**	**T**	**T**	**T**		Testis	**TBPL1**	**T**		**T**		Testis
**DNASE2B**	**T**		**T+**		Salivary gland	**HSPA1L**	**T**	**T**	**T**	**T**	Testis	**TCP11**	**T**	**T**	**T**		Testis
**ALPI**	**T**		**T+**		Small intestine	**LDHAL6B**	**T**	**T**	**T**	**T**	Testis	**TEX14**	**T**	**T**	**T**	*S+*	Testis
**GIP**	**T**		**T+**	T+	Small intestine	**LDHC**	**T**	**T**	**T**	**T**	Testis	**TNP1**	**T**	**T**	**T**		Testis
**SI**	**T**	**T**	**T+**	T+	Small intestine	**LOC81691**	**T**	**T**	**T**		Testis	**TPTE**	**T**	**T**	**T**		Testis
**ACTL7A**	**T**	**T**	**T**	*S+*	Testis	**NUP155**	**T**	**T**	**T**	*S+*	Testis	**TULP2**	**T**		**T**		Testis
**ACRV1**	**T**	**T**	**T**		Testis	**OAZ3**	**T**	**T**	**T**	**T+**	Testis	**PRSS16**	**T**		**T**	*S+*	Thymus
**ACTL7B**	**T**	**T**	**T**	**T**	Testis	**ODF1**	**T**		**T**		Testis	**CD1A**	**T**		**T**	*S*	Thymus
**ADAM2**	**T**	**T**	**T**	**T**	Testis	**ODF2**	**T**		**T**		Testis	**CD1B**	**T**		**T**	*S+*	Thymus
**AKAP4**	**T**	**T**	**T**		Testis	**PDHA2**	**T**		**T**		Testis	**CD1E**	**T**	**T**	**T**		Thymus
**ANKRD7**	**T**	**T**	**T**		Testis	**PHF7**	**T**	**T**	**T**	*S+*	Testis	**FBXL12**	**T**				Thymus
**APH1B**	**T**	**T**	**T**		Testis	**PIWIL1**	**T**	**T**	**T**	*S+*	Testis	**NFATC3**	**T**		**T**	*S+*	Thymus
**BRDT**	**T**	**T**	**T**	**T+**	Testis	**POLR2D**	**T**		**T**	*S+*	Testis	**RAG1**	**T**	**T+**	**T**		Thymus
**C19orf36**	**T**	**T**		**T**	Testis	**PPM1G**	**T**			**T+**	Testis	**TPO**	**T**		**T+**	**T**	Thyroid
**C1orf14**	**T**	**T**			Testis	**PRM1**	**T**	**T**		*S*	Testis	**TG**	**T**			**T**	Thyroid
**C20orf20**	**T**		**T**	**T**	Testis	**PRM2**	**T**	**T**	**T**		Testis	**SLC26A4**	**T**		**T**	*S+*	Thyroid
**CABYR**	**T**	**T**	**T+**		Testis	**PRND**	**T**	**T**	**T**	*S*	Testis						

The predicted results of the 191 genes (Pr) with best coverage and highest score are compared to TiGER (Ti), PaGenBase (PG) and HPA (HP). Agreements are shown in **bold** while disagreements in *italic*. ‘T’ is our predicted tissue, ‘S’ is another tissue from the databases. ‘T+’ means T and other identified. Empty white means: ubiquitously expressed, no tissue with strong support or no data (see Table S7 for more information).

Further, we find 11 out of 13 drug-targets and biomarkers with known tissue-specificity (described in the introduction and discussion). The two not found are predicted 2-selective including the correct tissue and results from stomach being missing in two of the sources. This is better than the databases where PaGenBase, TiGER and HPA find 6, 5 and 8, respectively (Table 7).

**Table 7:** Comparing the predicted results with TiGER, PaGenBase and HPA using a set of 13 drug targets and biomarkers that are known to have tissue-specific expression.

Target genes	Predicted	TiGER	PaGenBase	HPA	T
**ATP4A**	**T,S**		T,S,U	**T**	**Stomach**
**SCN5A**	**T**		**T**		**Heart**
**PNLIP**	**T**	**T**	**T,S**		**Pancreas**
**LIPF**	**T,S**	**T**		**T**	**Stomach**
**TPO**	**T**		**T,S**	**T**	**Thyroid**
**SLC5A2**	**T**	**T,S**	**T**	**T,S**	**Kidney**
**CRP**	**T**	**T**	**T**	**T**	**Liver**
**KLK3**	**T**	**T,S**	**T,S**	**T**	**Prostate**
**TNNT2**	**T**	**T**	**T**	**T**	**Heart**
**TG**	**T**	*S,U*	**T,S**	**T**	**Thyroid**
**SLC26A4**	**T**		**T**		**Thyroid**
**IYD**	**T**	*S*	**T**		**Thyroid**
**TSHR**	**T**	**T**	T,S,U	**T**	**Thyroid**

Agreements are shown in **bold** while disagreements in *italic*. ‘T’ is the target tissue, ‘S’ and ‘U’ are other identified tissues. Empty white means: ubiquitously expressed or no data (see Table S11, S12 and S13 for more information).

**Figure 6:**
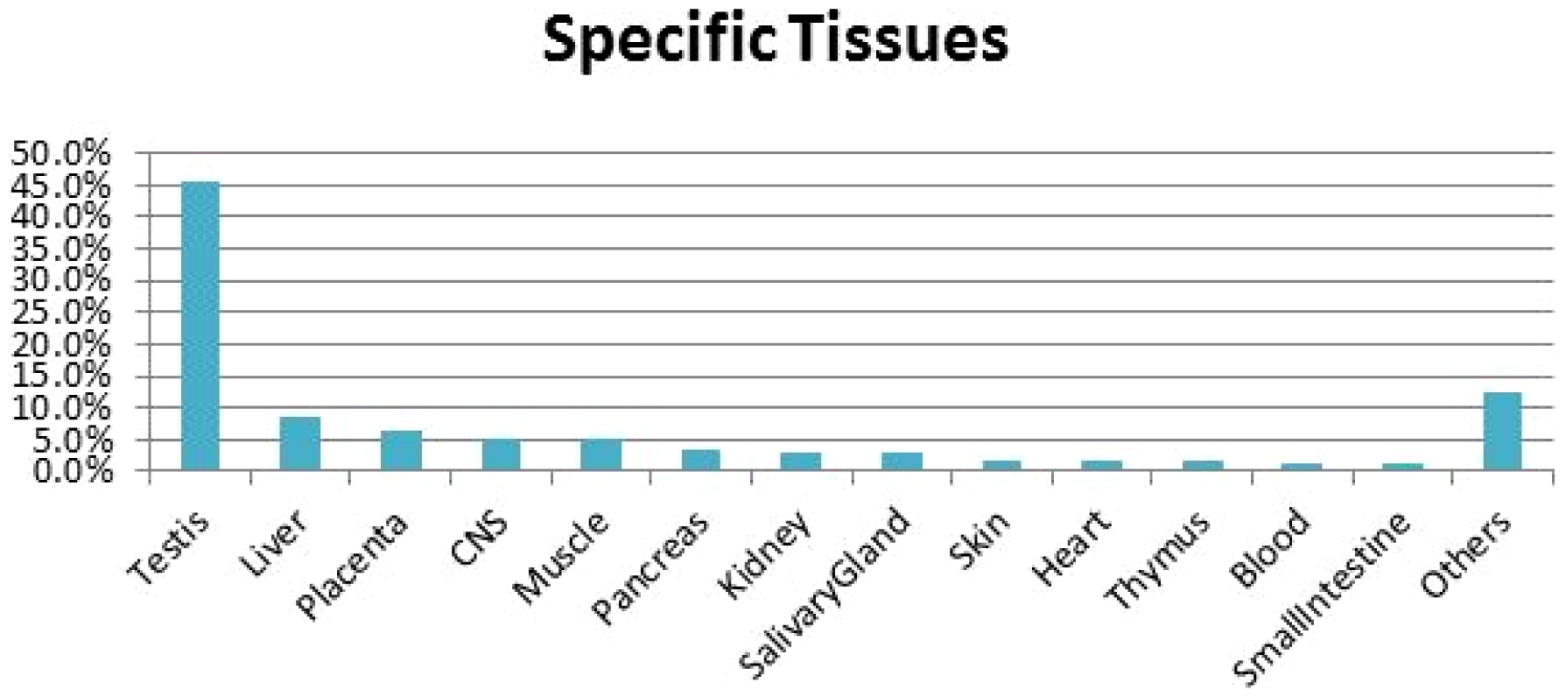
The ten most frequently occurring tissues among the 1685 specific genes. Many genes are, e.g., specifically expressed in the testis (about 41%). All Tissues are shown in Table S9.

We also analyzed which tissues were detected among the 1462 genes with strong support. The ten most frequently detected tissues are shown in Figure 6. We can see that testis, the male generative gland, is with about 46% the top candidate, followed by, liver, placenta, CNS, muscle, pancreas, kidney, salivary gland, skin, heart, thymus, blood, Small intestine, etc. Testis has previously been identified as the top candidate tissue for specific genes both in human [15] and mouse [25]. In the testis specific genes with highest score and coverage (Table 6) we find genes associated with the Gene Ontology term “spermatogenesis” (e.g. PRM1, PRM2, CCNA1, OAZ3, RPL39L and CCT6B) which is the biological process where germ-cells undergo division. Of the genes in the spermatogenesis process we predict 38 genes to be specific (score ≥0.5), 35 to testis, one to placenta and one to muscle.

The 31 2-selected genes with strong and medium-high support with full coverage were also analyzed (Table S8). The agreement between the different datasets is, not surprisingly, much lower than for the specific genes (Figure S1): 21% with TiGER, 32% with PaGenBase and 9% with HPA. The same numbers for partial agreement, i.e. at least one matching tissue, are: 61% with TiGER, 68% with PaGenBase and 69% for HPA. The pairs of tissues detected for the 2-selective genes are shown in the supplementary materials (Table S10 and Figure S2).

## Discussion

Data integration is the cornerstone of future informatics and will be crucial for effective drug discovery [5]. Two different approaches can be taken: 1) *Low-level integration* where the data is integrated before applying a method and, 2) *High-level integration* where the method is applied first and the results are aggregated. The first approach may sound more appealing but is often prevented due to the inability of combining data from different technologies and experiments. High-level integration has the advantage of more easily include and benefit from new datasets and technical platforms compared to adjusting a single expression compendium to accommodate new data. This is especially important as the field of expression analysis is expanding and evolving at a very high pace with respect to new studies and technologies. In high-level integration methods can be optimized for each data source separately. It can also be applied without access to the raw data as in *meta-analysis* of clinical trials where the aim is to identify general trends from several independent analyses.

The objective of this study was to evaluate the hypothesis of tissue-specificity over multiple data sources using a high-level approach. The fundamental reasoning is that if many sources (on average) point to the same conclusion, we can be more confident. Two steps are evaluated: 1) Optimize the performance of an individual method with respect to a dataset, 2) Combine the outcome from several methods. The first step was achieved by adjusting the parameters using training genes and with the rule-based score (i.e. the inner-score). The second step was achieved with the total score which is basically the average over all sources. The result is a list of tissue-specific genes which is assessed by two values: support and coverage. The first tells us how convincing the identification was (regardless of the number of sources), and the latter how consistent the identification was over the sources.

In order to qualify for strong support (score = 1) and the full coverage (*4 out of 4*, Table 5), a gene must be predicted specific by all selected method/dataset pairs. In addition it must be represented on all platforms and expressed in a tissue group found in each dataset. Many specific genes will never qualify for this strict requirement but will still have strong support. For example, the 1011 specific genes predicted by only one method/dataset pair (Table 5) all have a strong signal but are only found in one dataset. This is expected since the datasets have large differences both in size and gene scope.

It is inherently difficult to validate our approach by comparing the outcome with the “true” picture since this is not known despite many investigations of gene (and protein) expressions. Most often such investigations are focused on the tissue of interest while the majority of organs are omitted or subjected to less detailed investigation. Even interrogative large omics datasets suffer from incomplete tissue panels or too low granularity/resolution or platform-dependent lack of probes. Thus, comparing our data to that of individual datasets is of little help to detect false negative and positive results. True positive results should however be possible to readily confirm. E.g., high scores for tissue-selectivity were obtained for the genes mentioned in the introductory part. These were predicted to be specific with high support for SCN5A in the heart (flecainide for prevention of arrhythmias), high support for PNLIP and strong support for LIPF in the small intestine and stomach, respectively (orlistat for reducing lipid uptake from the gut), strong support for TPO in the thyroid gland (methimazole for hyperthyroidism), strong support for SLC5A2 in kidney, medium support for CRP in the liver, high support for KLK3 in the prostate, and strong support for TNNT2 in the heart. The low score for ATP4A in the stomach (omeprazole for neutralizing gastric acid) is caused by the lack of stomach in the GNF1H dataset.

Another attempt to validate our result is to look for functional rather than platform/technique-dependent supporting observations: For instance, diseases specific to the thyroid gland are caused by mutations in or by autoimmune reactions to the thyroid-specific genes. Our predictions give strong support for thyroid-specific expression of the genes TPO (strong support) and TG (strong support) encoding proteins malfunctions of which cause Hashimoto's thyroiditis (OMIM [26] ID 140300). Thyroglobulin encoded by TG (strong support) is also well known for its binding of and thyroid gland-specific storage of iodine. Autoimmune reactions towards TSHR (strong support) cause Graves disease (OMIM ID 275000). Mutations in SLC26A4 (strong support) or IYD (strong support) cause Pendred syndrome (OMIM ID 274600) or iodotyrosine deiodinase deficiency, respectively (OMIM ID 274800).

New RNA-seq technology [27] will most likely increase the precision of tissue expression data. The power of this technique is its dynamic range and that it measures what is there and not what is asked for. However, this detailed information will be most valuable when gathered from a complete and extensive tissue panel if the aim is to identify biomarkers and tissue-specific drug targets. The approach described in this work is equally important for other type of omic data including RNA-seq as well as for the exploitation of micro-RNAs. This recently highlighted class of nucleic acids is expected to contribute to the options for both targets and biomarkers. Their property of being stable and appearing in a cell-free form in blood has already been shown to reflect the condition of the source organ [28], [29], [30]. Several data sources describing the tissue distribution of most of the around 800 micro-RNAs expressed in man are available [31], [32].

In addition to tissue-specific gene expressions, we have identified 2-selective genes and could argue also for the value of identifying 3-selective genes, etc. The motive is that drugs might target two tissues with beneficial (or one neutral) effects or that the drug may access only one organ. An example of the latter situation is drugs that target an organ in the periphery but do not cross the blood-brain barrier (e.g. clopidogrel and ticagrelor blocking P2RY12 on platelets but not neuronal tissue in brain and spinal cord). An example of 2- or 3-selective expression is provided by NPC1L1 which is expressed in the liver and intestine and is the target for the drug ezetimibe. These two examples also highlight the issue of granularity, coverage, and vocabulary for defining and grouping tissue. For example, P2RY12 scores selective for spinal cord and central nervous system (CNS) but is also expressed on platelets which are the target cells for the drug and not included in the tissue panels of any of the datasets. Spinal cord and CNS could with a lower resolution vocabulary be grouped as neuronal tissue. In the case of NPC1L1 the selective expression in duodenum and jejunum could be grouped and classified as small intestine-specific (in addition to liver-specific).

## Conclusion

Identifying tissue-specific genes is a difficult problem with many pitfalls. Despite these we are convinced that our analysis provides valuable information when formulating and testing new biological hypotheses. We encourage future method development and anticipate that our gene sets will be valuable as a benchmark for new technology data.

## Supporting Information

Figure S1 The agreement of the 31 2-selective genes with strong or medium-high support and highest coverage, based on our predictions, to TiGER, PaGenBase (TiSGeD) and HPA. No info means missing data.
(EPS)

Figure S2 The frequently co-occurring tissues among the 214 predicted 2-selected genes with strong support.
(EPS)

Table S1 Grouping of functionally and literary similar tissues.
(DOCX)

Table S2 Predicted tissues on mixed training genes.
(DOCX)

Table S3 Predicted tissues on kidney training genes.
(DOCX)

Table S4 Predicted tissues on muscle training genes.
(DOCX)

Table S5 Predicted tissues on prostate and lung training genes.
(DOCX)

Table S6 Predicted tissues on liver training genes.
(DOCX)

Table S7 Comparison of Specific Genes.
(DOCX)

Table S8 Comparison of 2-Selective Genes.
(DOCX)

Table S9 The frequency of detected tissues.
(DOCX)

Table S10 The frequency of pairs of detected tissues.
(DOCX)

Table S11 Comparision of the predicted results of the 13 drug target genes with TiGER, PaGenBase and HPA.
(DOCX)

Table S12 Comparision of the predicted results of the 13 drug target genes with TiGER, PaGenBase and HPA (GeAZr data excluded).
(DOCX)

Table S13 Shows the predicted tissue for each of the datasets for the 13 drug target genes.
(DOCX)

Text S1 Additional information around method, data and results.
(DOCX)

Text S2 A list of all genes with corresponding score and coverage.
(XLSX)
