# Supplementary material for: Combining Evidence of Preferential Gene-Tissue Relationships from Multiple Sources
Source: PLoS One. 2013 Aug 12;8(8):e70568. doi: 10.1371/journal.pone.0070568 (PMC3741196; doi:10.1371/journal.pone.0070568)
Supplement: Table S1 — Grouping of functionally and literary similar tissues. (DOCX) [file pone.0070568.s003.docx]

Table S1 Grouping of functionally and literary similar tissues. The original terms from data sets are mapped onto the new terms. The Samples in dataset column shows the numbers of terms in each dataset that was mapped to this term, the order is GNF1H, GeAZr, GDS3113, GSE7307.

| **Tissue name after grouping** | **Number of tissue terms in data sets** | **Original tissue names from 5 data sets** |
| --- | --- | --- |
| **Adipocyte** | 1,3,0,3 | Adipose Tissue, Adipose Tissue Omental, Adipose Tissue Subcutaneous, Adipose tissue of breast |
| **Adrenal** | 2,2,1,1 | Adrenal Cortex, Adrenal Gland |
| **Apendix** | 1,1,0,0 | Apendix |
| **Articular surface of bone** | 0,1,0,0 | Articular surface of bone |
| **Bile** | 0,2,0,0 | Common bile duct, Gallbladder |
| **Bone Structure** | 0,1,0,0 | Bone Structure |
| **Bone Marrow** | 1,0,1,1 | Bone Marrow |
| **Breast** | 0,1,0,1 | Breast |
| **Bronchus** | 0,1,0,1 | Bronchus |
| **Cerebellum** | 2,1,0,3 | Cerebellar Hemisphere, Cerebellar Vermis, Cerebellum, Cerebellum Peduncles |
| **Cervix** | 0,1,0,1 | Cervix |
| **Colon** | 1,1,1,2 | Colon, Colon Cecum |
| **Clorectal adenocrina** | 1,0,0,0 | Clorectaladenocrina |
| **CNS** | 16,25,1,30 | Accumbens, Amygdala, Amygdaloid nucleus, Brain, Caudate, Caudate nucleus, Cerebral Cortex, Cingulate gyrus, Cingulate cortex, colon, Corpus callosum, Entorhinal cortex, Frontal cortex, Frontal Lobe, Globus pallidus, Gloubus Pallidum External, Gloubus Pallidum Internal, Hippocampus, Hypothalamus, Locus ceruleus, Medulla, Medulla oblongata, Midbrain, Nodose Nucleus, Nucleus Accumbens, Nucleus basalis of Meynert, Occipital cortex, Occipital Lobe, Olfactory Bulb, Parietal cortex, Parietal Lobe, Pituitary, Pituitary gland, Pons, Prefrontal Cortex, Pulvinar, Putamen, Red nucleus, Substantia nigra, Substantia Nigra Pars Compacta, Substantia Nigra Reticulata, Subthalamic nucleus, Temporal cortex, Temporal Lobe, Thalamus, Thalamus Lateral Nuclei, Thalamus Subthalamic Nucleus, Vestibular Nuclei Superior, White matter of occipital lobe |
| **Endometrium** | 0,1,0,1 | Endometrium |
| **Epididymis** | 0,1,0,0 | Epididymis |
| **Esophagus** | 0,1,0,1 | Esophagus |
| **Fallopian tube** | 0,1,0,1 | FallopianTube |
| **Heart** | 2,5,1,4 | Atrioventricular Node, Heart, Heart Atrium, Heart Ventricle, Left atrium, Left ventricle, Pericardium, Right atrium, Right ventricle |
| **Kidney** | 1,2,1,3 | Bladder, Kidney, Kidney Cortex, Kidney Medulla |
| **Larynx** | 0,1,0,0 | Larynx |
| **Leukemia** | 3,0,0,0 | Leukemia chronic Myelogenous K-562, Leukemia promyelocytic-HL-60, Leukemialymphoblastic MOLT-4 |
| **Leukocyte** | 0,1,0,0 | leukocyte |
| **Liver** | 1,2,1,1 | Hepatic duct, Liver |
| **Lung** | 1,1,1,1 | Lung |
| **Lymph** | 4,1,1,1 | 721 B lymphoblasts, Lymphnode, Lymphoma burkittsDaudi, Lymphoma burkittsRaji |
| **Mammary gland** | 0,0,1,1 | Mammary gland |
| **Meniscus joint** | 0,1,0,0 | MeniscusJoint |
| **Muscle** | 3,2,1,2 | Cardiac myocytes, Skeletal muscle, Smooth muscle, Deltoid muscle |
| **Myometrium** | 0,1,0,1 | Myometrium |
| **Nerve** | 4,1,0,2 | Ciliary ganglion, Dorsal root ganglion, Superior cervical ganglion, Trigeminal ganglion |
| **Omentum** | 0,1,0,0 | Omentum |
| **Ovary** | 1,1,1,1 | Ovary |
| **Pancreas** | 2,1,1,1 | Pancreas, PancreaticIslet |
| **Pineal** | 2,0,0,0 | Pineal day, Pineal night |
| **Placenta** | 1,1,1,1 | Placenta |
| **Prostate** | 1,1,1,2 | Prostate, Prostate Gland |
| **Rectum** | 0,1,0,0 | Rectum |
| **retina** | 1,0,1,0 | Retina |
| **Salivary gland** | 1,2,1,1 | Parotid gland, Salivary gland |
| **Seminal vesicle** | 0,1,0,0 | Seminal vesicle |
| **Skin** | 1,1,1,1 | Skin |
| **Small intestine** | 1,4,1,4 | Duodenum, Ileum, Jejunum, Small intestine, Small Intestine BD |
| **Soft tissue** | 0,1,0,0 | Soft tissue |
| **SpinalCord** | 1,1,1,1 | SpinalCord |
| **Spleen** | 0,1,1,1 | Spleen |
| **Stomach** | 0,1,0,4 | Stomach, Stomach Cardiac, Stomach Fundus, Stomach Pyloric |
| **Tendon** | 0,1,0,0 | Tendon |
| **Testis** | 5,1,1,1 | Testis, Testis germ cell, Testis intersitial, Testis leydig cell, Testis seminiferous tubule |
| **Thymus** | 1,1,1,1 | Thymus |
| **Thyroid** | 1,1,1,1 | Thyroid |
| **Tongue** | 1,1,0,3 | Tongue, Tongue Main Corpus, Tongue Superior With Papillae |
| **Tonsil** | 1,1,1,1 | Tonsil |
| **Trachea** | 1,1,1,1 | Trachea |
| **Ureter** | 0,1,0,0 | Ureter |
| **Urethra** | 0,1,0,1 | Urethra |
| **Uterus** | 2,1,1,1 | Uterus, UterusCorpus |
| **Vagina** | 0,1,0,1 | Vagina |
| **Vas deferens** | 0,1,0,0 | VasDeferens |
| **Vessel** | 0,7,0,3 | Abdominal aorta, Aorta, Artery, Ascending aorta, Blood vessel, Coronary artery, Vein |
| **Vulva** | 0,1,0,1 | Vulva |
| **WholeBlood** | 1,0,0,0 | WholeBlood |
