# Supplementary material for: Combining Evidence of Preferential Gene-Tissue Relationships from Multiple Sources
Source: PLoS One. 2013 Aug 12;8(8):e70568. doi: 10.1371/journal.pone.0070568 (PMC3741196; doi:10.1371/journal.pone.0070568)
Supplement: Table S2 — Predicted tissues on mixed training genes. (DOCX) [file pone.0070568.s004.docx]

Table S2 – Predicted tissues after optimization on the mixed set of training genes. ‘-‘ indicates that no specific tissue was identified. The methods applied are ROKU SPM (RS), Decision Function (DF), Bayes Factor (BF). Gray color shows that the detected result is exactly same as the HugeIndex database, and the red color shows that the detected result is partially same as the HugeIndex database.

| **Data** | **GNF1H** | | | | **GeAZr** | | **GSE7307** | | | **GDS3113** | | | **Tissue Names** |
| --- | --- | --- | --- | --- | --- | --- | --- | --- | --- | --- | --- | --- | --- |
| **Method** | **RS** | **DEC** | | **BF** | **RS** | **DEC** | **RS** | **DEC** | **BF** | **RS** | **DEC** | **BF** |  |
| **Gene** | **Detected tissues** | | | | | | | | | | | |  |
| **FXYD2** | T | | T | T,S | T | T | T | - | - | T,S | - | - | T=Kidney, S=Salivary gland |
| **PAX8** | T | | T | T | T | T | - | - | T | T | T | - | T=Thyroid |
| **HABP2** | T | | T | T | T | T | T | T | T | T,S | T | - | T=Liver, S=Muscle |
| **SAA4** | T | | T | T | T | T | T | T | T | T | T | T | T=Liver |
| **CPN2** | T | | T | T | T | T | T | T | T | T | T | T | T=Liver |
| **ASGR1** | T | | T | T | T | T | T | T | T | T,S | T | T | T=Liver, S=Bone marrow |
| **LIPC** | T | | T | T | T | T | T | T | T | - | T | - | T=Liver |
| **SFTPC** | T | | T | T | T | T | T | T | T | T | T | T | T=Lung |
| **SFTPB** | T | | T | T | T | T | T | T | T | T | T | T | T=Lung |
| **KLK2** | T | | T | T | T | T | T | T | T | T,S | T | T,S | T=Prostate, S=Salivary gland |
| **ACPP** | T | | T | T | T | T | T | T | T | T | T | T | T=Prostate |
| **CA3** | T | | T | T,S | T | T | T,S | - | T | - | T | T | T=Thyroid, S=Muscle |
| **APOC3** | T | | T | T,S | T | T | T,S | T,S | T | T,S | T,S | T,S | T=Liver, S=Small intestine |
| **KLK3** | T | | T | T | T | T | T | T | T | T | T | - | T=Prostate |
| **LOR** | T | | T | T | T | T | T,S | T,S | - | T,S | T | T | T=Skin, S=Thymus |
| **TPMT** | T | | T | - | T | - | - | - | - | T,S | T,S | - | T=Heart, S=Muscle |
| **ITIH2** | T | | T | T | T | T | T | T | T | T,S | T | T,S | T=Liver, S=Spinal cord |
| **DPEP1** | T,S | | T,S | - | - | - | T | - | - | T,S | - | - | T=Kidney, S=Pancreas |
| **TTR** | - | | - | - | T | T | T,S | - | T,S | T,S | - | - | T=Liver, S=Retina |
| **USP13** | T | | T | T | T | T | - | - | T | T | T,S | - | T=Muscle, S=Heart |
| **VIM** |  | |  |  |  |  |  |  |  |  |  |  |  |
| **RPL41** |  | |  |  |  |  |  |  |  |  |  |  |  |
| **E2F4** |  | |  |  |  |  |  |  |  |  |  |  |  |
| **XPOT** |  | |  |  |  |  |  |  |  |  |  |  |  |
| **UQCRH** |  | |  |  |  |  |  |  |  |  | T |  | T=Heart |
| **SEPW1** |  | |  |  |  |  |  |  |  |  |  |  |  |
| **YWHAQ** |  | |  |  |  |  |  |  |  |  |  |  |  |
| **PSD** |  | |  | T |  |  |  |  |  | T |  |  | T=CNS |
| **PSMB5** |  | |  |  |  |  |  |  | T |  |  |  | T=Skeletal muscle |
| **CFL1** |  | |  |  |  |  |  |  |  |  |  |  |  |
