# Supplementary material for: Combining Evidence of Preferential Gene-Tissue Relationships from Multiple Sources
Source: PLoS One. 2013 Aug 12;8(8):e70568. doi: 10.1371/journal.pone.0070568 (PMC3741196; doi:10.1371/journal.pone.0070568)
Supplement: Table S5 — Predicted tissues on prostate and lung training genes. (DOCX) [file pone.0070568.s007.docx]

Table S5 - Predicted tissues after optimization on the Prostate and Lung set of training genes (after consensus vote). Empty white indicates that no specific tissue was identified. Gray color shows that the detected result is exactly same as the HugeIndex database, and the red color shows that the detected result is partially same as the HugeIndex database.

| **DATASET** | **GNF1H** | **GeAZr** | **GSE7307** | | **GDS3113** | | | **TISSUE ANNONTATION** | |
| --- | --- | --- | --- | --- | --- | --- | --- | --- | --- |
| **GENE** | **CONSENSUS VOTE** | | | | | | |  |  |
| **HOXB13** | T | T | | T,S | | T | T=Prostate, S=Colon | |  |
| **ARG2** | T | T | |  | |  | T=Prostate | |  |
| **NPY** | T | T | | T | |  | T=Prostate | |  |
| **SEMG1** | T | S | | T | | T | T=Prostate, S=Vas Deferens | |  |
| **MARCO** | T |  | |  | |  | T=Lung | |  |
| **CLDN18** | T | S | | T,S | | T | T=Lung, S=Stomach | |  |
| **FCN3** | S |  | | T,S | | T | T=Lung ,S=Liver | |  |
| **DUSP1** | T |  | |  | |  | T=Lung | |  |
| **PGC** | T | S | | S | | T | T=Lung, S=Stomach | |  |
| **LAMP3** | T | T | |  | |  | T=Lung | |  |
| **RPL19** |  |  | |  | |  |  | |  |
| **CD63** |  |  | |  | |  |  | |  |
| **WARS** |  |  | |  | |  |  | |  |
| **UBA52** |  |  | |  | |  |  | |  |
| **HLA-E** |  |  | |  | |  |  | |  |
| **RPL23** |  |  | |  | |  |  | |  |
| **RPL17** |  |  | |  | |  |  | |  |
| **FLNA** |  |  | |  | |  |  | |  |
| **RPL35A** |  |  | |  | |  |  | |  |
| **EEF2** |  |  | |  | |  |  | |  |
