# Supplementary material for: Combining Evidence of Preferential Gene-Tissue Relationships from Multiple Sources
Source: PLoS One. 2013 Aug 12;8(8):e70568. doi: 10.1371/journal.pone.0070568 (PMC3741196; doi:10.1371/journal.pone.0070568)
Supplement: Table S7 — Comparison of Specific Genes. (DOCX) [file pone.0070568.s009.docx]

Table S7 - Comparison of Specific Genes.

Compare results of 191 specific genes with the best coverage and highest score to TiGER, PaGenBase and HPA. Gray shows the exact agreement in the corresponding database, red shows partially agreement, and absence and disagreement remain white. ‘\’ means the gene is not found in the database and ‘-‘ means this gene is not specific.

|  | | **PREDICTED** | **TIGER** | **PAGENBASE** | **HPA** |  |  |
| --- | --- | --- | --- | --- | --- | --- | --- |
| **CYP17A1** | | Adrenal | Kidney | Adrenal | Several tissues with strong, Adrenal (Strong) |  |  |
| **FDXR** | | Adrenal | Cervix | Adrenal | Many tissues with strong, Adrenal (Strong) |  |  |
| GSTA4 | | Adrenal | - | Adrenal | No expression data |  |  |
| **HSD3B2** | | Adrenal | - | Adrenal, Ovary | No expression data |  |  |
| NOV | | Adrenal | - | Adrenal | Several tissues with strong, Adrenal (Strong) |  |  |
| **LY6H** | | CNS | CNS | CNS | No expression data |  |  |
| CAMK1G | | CNS | - | CNS | Many tissues with strong, CNS (Strong) |  |  |
| HTR2A | | CNS | \ | CNS, Breast | Several tissues with strong, CNS (Strong) |  |  |
| **TNNT2** | | Heart | Heart | Heart | Heart (Strong) |  |  |
| **MYL7** | | Heart | Heart | Heart | Heart (Strong) |  |  |
| **MYBPC3** | | Heart | Heart | Heart | Heart (Strong) |  |  |
| **MYL4** | | Heart | Heart | Heart | Several tissues with strong, Heart (Strong) |  |  |
| NPPA | | Heart | Heart | Heart | No expression data |  |  |
| NPPB | | Heart | Heart | Heart | No expression data |  |  |
| **SLC12A3** | | Kidney | Cervix, Kidney | Kidney | Several tissues with strong, Kidney (Moderate) |  |  |
| **SLC34A1** | | Kidney | Kidney | Kidney | Several tissues with strong, Kidney (Strong) |  |  |
| **SLC22A6** | | Kidney | Kidney | Kidney | No expression data |  |  |
| CUBN | | Kidney | Kidney | Kidney | No expression data |  |  |
| KL | | Kidney | Kidney | Kidney | Many tissues with strong, CNS (Strong) |  |  |
| NPHS2 | | Kidney | Kidney | Kidney | Kidney (Strong) |  |  |
| SLC22A2 | | Kidney | Kidney | Kidney | Several tissues with strong, Kidney (Moderate) |  |  |
| SLC5A2 | | Kidney | Kidney, Testis | Kidney | Several tissues with strong, Kidney (Strong) |  |  |
| **CYP1A2** | | Liver | \ | Liver | Liver (Strong) |  |  |
| **AGXT** | | Liver | Liver | Liver | Liver (Strong), Testis (Strong) |  |  |
| **AHSG** | | Liver | Liver | Liver | No Strong |  |  |
| AKR1D1 | | Liver | Liver | Liver | No expression data |  |  |
| **ANG** | | Liver | Liver | Liver | No expression data |  |  |
| APOB | | Liver | Liver, Small intestine, Spleen | Liver | Kidney (Strong) |  |  |
| **APOC2** | | Liver | Liver | Liver | Kidney (Strong), |  |  |
| APOF | | Liver | \ | Liver | No expression data |  |  |
| **APOH** | | Liver | Liver | Liver | Several tissues with strong, Liver (Moderate) |  |  |
| **ASGR1** | | Liver | Liver | Liver | Liver (Strong) |  |  |
| ATF5 | | Liver | - | Liver | Many tissues with strong, Liver (Moderate) |  |  |
| BAAT | | Liver | Liver | Liver | Several tissues with strong, Liver (Strong) |  |  |
| C2 | | Liver | Soft tissue | Liver | No Strong | | |
| C8A | | Liver | Liver | Liver | No expression data | | |
| C8B | | Liver | Liver | Liver | No Strong, Liver (Moderate) | | |
| **C8G** | | Liver | Liver, Stomach | Liver | Several tissues with strong, Stomach (Strong), Liver (Moderate) | | |
| C9 | | Liver | Liver | Liver | Several tissues with strong, Liver (Moderate) | | |
| CPN2 | | Liver | \ | Liver | Many tissues with strong, Liver (Moderate) | | |
| **CYP2A6** | | Liver | Liver | Liver | No expression data | | |
| **CYP2A7** | | Liver | Liver, Mammary gland | Liver, Prostate | Liver (Strong) | | |
| CYP2C8 | | Liver | Liver | Liver | Liver (Strong) | | |
| CYP2C9 | | Liver | Liver | Liver | Liver (Strong) | | |
| **CYP2E1** | | Liver | Liver | Liver | Liver (Strong) | | |
| **F12** | | Liver | Liver, Stomach | Liver | No Strong | | |
| **F2** | | Liver | - | Liver | Several tissues with strong | | |
| F7 | | Liver | \ | Liver | Many tissues with strong, Liver (Moderate) | | |
| F9 | | Liver | Liver | Liver | Several tissues with strong, Liver (Weak) | | |
| **FGA** | | Liver | Liver | Liver | Several tissues with strong | | |
| **FGB** | | Liver | Liver | Liver, Kidney | Several tissues with strong, Liver(Strong) | | |
| **FGG** | | Liver | Liver | Liver | No Strong | | |
| **GC** | | Liver | Liver | Liver | Several tissues with strong, Liver(Strong) | | |
| GCKR | | Liver | \ | Liver | No expression data | | |
| **HABP2** | | Liver | Liver, Kidney, Muscle | Liver | Several tissues with strong, Liver (Moderate) | | |
| **HAMP** | | Liver | - | Liver | No expression data | | |
| HAO1 | | Liver | Liver | Liver | Liver (Strong) | | |
| **HGFAC** | | Liver | \ | Liver | Several tissues with strong, Liver (Moderate) | | |
| **HPR** | | Liver | Liver | Liver | Several tissues with strong, Liver (Strong) | | |
| **HPX** | | Liver | Liver | Liver | No Strong, Liver (Weak) | | |
| INHBE | | Liver | Muscle | Liver | Several tissues with strong, Liver (Strong) | | |
| **ITIH1** | | Liver | Liver | Liver | Several tissue strong, Liver (moderate) | | |
| ITIH2 | | Liver | Liver, Spleen | Liver | Many tissues with strong, Liver (Strong) | | |
| **ITIH3** | | Liver | Liver | Liver | Several tissues with strong, Liver(Moderate) | | |
| KLKB1 | | Liver | Liver | Liver | Many tissues with strong, Liver (Strong) | | |
| LPA | | Liver | - | Liver | No Strong, Liver (Weak) | | |
| MASP2 | | Liver | Liver | Liver | Several tissues with strong, Liver(Moderate) | | |
| **MAT1A** | | Liver | Liver | Liver | No expression data | | |
| MBL2 | | Liver | \ | Liver | Liver (Strong) | | |
| NR1I3 | | Liver | Kidney, Liver | Liver | No Strong | | |
| PON1 | | Liver | Liver | Liver | Several tissues with strong, Liver (Strong) | | |
| **PROC** | Liver | | Liver | Liver | Several tissues with strong, Liver (Moderate) | |  |
| **SAA4** | Liver | | Liver | Liver | No Strong, Liver (Weak) | |  |
| SERPINA10 | Liver | | Liver | Liver | CNS and Stomach Strong | |  |
| SERPINA7 | Liver | | Liver | Liver | No Strong, Liver (Moderate) | |  |
| **SERPINC1** | Liver | | Liver | Liver | Several tissues with strong, Liver (Moderate) | |  |
| **SERPIND1** | Liver | | Liver | Liver | No Strong | |  |
| **SERPINF2** | Liver | | Liver, Kidney | Liver | Many tissues with strong, Liver (Strong) | |  |
| SLC10A1 | Liver | | Liver | Liver | Several tissues with strong, Liver (Moderate) | |  |
| **SLC22A1** | Liver | | Liver | Liver, Ovary | Uterine Cervix (Strong), Liver (Moderate) | |  |
| **SPP2** | Liver | | Liver | Liver | No Strong | |  |
| TDO2 | Liver | | Liver | Liver | Several tissues with strong, Liver (Weak) | |  |
| **VTN** | Liver | | Liver | Liver | No expression data | |  |
| **SFTPC** | Lung | | Lung | Lung | Lung (Strong) | |  |
| **AGER** | Lung | | Lung | Lung | Several tissues with strong, Lung (Strong) | |  |
| **SFTPB** | Lung | | Lung | Lung | No strong, Lung (Moderate) | |  |
| **SFTPD** | Lung | | Lung | Lung | Lung (Strong), Colon (Strong) | |  |
| **APOBEC2** | Muscle | | Pancreas | Muscle | Pancreas (Strong), Muscle(Moderate) | |  |
| **MYOZ1** | Muscle | | Muscle | Muscle, Prostate | Muscle (Strong) | |  |
| AMPD1 | Muscle | | Muscle | Muscle, Thyroid | Several tissues with strong, Muscle (Strong) | |  |
| MYF6 | Muscle | | Heart, Muscle | Muscle | No expression data | |  |
| NEB | Muscle | | heart, larynx, muscle, tongue | Muscle, Thyroid | Muscle (Strong) | |  |
| RPL3L | Muscle | | Muscle | Muscle, Heart | Liver (Strong), Muscle (Moderate) | |  |
| **PSG2** | Placenta | | Placenta | Placenta | No expression data | |  |
| **ADAM12** | Placenta | | Placenta | Placenta, Adipose | Several tissues with strong, Placenta (Strong) | |  |
| **CYP19A1** | Placenta | | Placenta | Placenta, Ovary | Placenta (Strong), CNS(Strong) | |  |
| **EBI3** | Placenta | | Placenta | Placenta | Several tissues with strong, Placenta (Moderate) | |  |
| **GCM1** | Placenta | | Placenta | Placenta, Kidney, Adrenal | Placenta (Strong) | |  |
| **HMGB3** | Placenta | | - | Placenta | No expression data | |  |
| **HSD17B1** | Placenta | | Placenta | Placenta | Placenta (Strong) | |  |
| INSL4 | Placenta | | Placenta | Placenta, Thyroid | No expression data | |  |
| LGALS13 | Placenta | | Placenta | Placenta, Testis | Several tissues with strong, Placenta (Moderate) | |  |
| **LGALS14** | Placenta | | Placenta | Placenta, CNS | No expression data | |  |
| MAGEA8 | Placenta | | Placenta, Spleen | Placenta | Planceta (Strong), Testis (Strong) | |  |
| **MAN1C1** | Placenta | | Placenta | Placenta | Placenta (Strong), Testis (Strong) | |  |
| **PLAC1** | Placenta | | Placenta | Placenta | No expression data | |  |
| **PSG11** | Placenta | | Placenta | Placenta | Several tissues with strong, Placenta (Moderate) | |  |
| **PSG3** | Placenta | | Placenta | Placenta, Testis | Several tissues with strong, Placenta (Moderate) | |  |
| **PSG4** | Placenta | | Placenta | Placenta | Several tissues with strong, Placenta (Moderate) | |  |
| **PSG5** | Placenta | | Placenta | Placenta, Adipose | Several tissues with strong, Placenta (Moderate) | |  |
| **PSG6** | Placenta | | Placenta | Placenta, CNS | Several tissues with strong, Placenta (Moderate) | |  |
| **PSG7** | Placenta | | Placenta | Placenta, Liver | No expression data | |  |
| **PSG9** | Placenta | | Placenta | Placenta | Several tissues with strong, Placenta (Moderate) | |  |
| **RASA1** | Placenta | | Bone marrow | Placenta | Several tissues with strong, Placenta (Strong) | |  |
| SIGLEC6 | Placenta | | Placenta | Placenta | Planceta (Strong) | |  |
| **KLK2** | Prostate | | Prostate, Nerve | Prostate, Testis | Prostate (Strong) | |  |
| PAK1IP1 | Prostate | | Bone | Prostate | Many tissues with strong, Placenta (Strong) | |  |
| TGM4 | Prostate | | Prostate | Prostate | Prostate (Strong), CNS (Strong) | |  |
| **CST4** | Salivary gland | | Bladder, Colon | Salivary gland | No expression data | |  |
| **CST5** | Salivary gland | | \ | Salivary gland, Kidney | No Strong, Salivary gland (Moderate) | |  |
| **CA6** | Salivary gland | | \ | Salivary gland, Lung | Salivary gland (Strong) | |  |
| **CST2** | Salivary gland | | - | Salivary gland | Salivary gland (Strong) | |  |
| DNASE2B | Salivary gland | | Lung | Salivary gland, Lymph | No expression data | |  |
| ALPI | Small intestine | | \ | Small intestine, Kidney | No expression data | |  |
| GIP | Small intestine | | \ | Small intestine, Colon, Testis | Several tissues with strong, Small intestine (Strong) | |  |
| SI | Small intestine | | Small intestine | Small intestine, Colon, Testis | Several tissues with strong, Small intestine (Strong) | |  |
| **ACTL7A** | Testis | | Testis | Testis | Several tissues with strong, Testis (Moderate) | |  |
| ACRV1 | Testis | | Testis | Testis | No Strong, Testis (Moderate) | |  |
| **ACTL7B** | Testis | | Testis | Testis | Testis (Strong) | |  |
| ADAM2 | Testis | | Testis | Testis | Testis (Strong) | |  |
| **AKAP4** | Testis | | Testis | Testis | No Strong, Testis (Moderate) | |  |
| **ANKRD7** | Testis | | Testis | Testis | Several tissues with Strong, Testis (Moderate) | |  |
| **APH1B** | Testis | | Testis | Testis | No expression data | |  |
| **BRDT** | Testis | | Testis | Testis | Many tissues with strong, Testis (Strong) | |  |
| **C19orf36** | Testis | | Testis | \ | Testis (Strong) | |  |
| C1orf14 | Testis | | Testis | \ | No expression data | |  |
| C20orf20 | Testis | | \ | Testis | Testis (Strong) | |  |
| **CABYR** | Testis | | Testis | Testis, Breast | Testis (Moderate) | |  |
| **CCIN** | Testis | | Testis | Testis | Many tissues with strong, Testis (Strong) | |  |
| **CCNA1** | Testis | | Bone marrow, Testis | Testis | No expression data | |  |
| **CCT6B** | Testis | | Testis | Testis | No expression data | |  |
| **COIL** | Testis | | Testis | Testis | Several tissues with strong, Testis (Strong) | |  |
| **CSNK2A2** | Testis | | - | Testis | No expression data | |  |
| CYLC2 | Testis | | \ | Testis | No expression data | |  |
| **DDX4** | Testis | | Testis | Testis | Testis (Strong) | |  |
| DKKL1 | Testis | | Testis | Testis | Testis (Strong) | |  |
| DMRT1 | Testis | | Testis | Testis | Gallbladder (Strong), Testis (Moderate) | |  |
| FBXO24 | Testis | | Testis | Testis | Many tissues with strong, Testis (Strong) | |  |
| GK2 | Testis | | Testis | Testis | No expression data | |  |
| **HSPA1L** | Testis | | Testis | Testis | Testis (Strong) | |  |
| LDHAL6B | Testis | | Testis | Testis | Testis (Strong) | |  |
| **LDHC** | Testis | | Testis | Testis | Testis (Strong) | |  |
| **LOC81691** | Testis | | Testis | Testis | No expression data | |  |
| **NUP155** | Testis | | Testis | Testis | Several tissues with strong, Testis (Moderate) | |  |
| **OAZ3** | Testis | | Testis | Testis | Many tissues with strong, Testis (Strong) | |  |
| **ODF1** | Testis | | \ | Testis | No expression data | |  |
| **ODF2** | Testis | | - | Testis | No strong, Testis (Weak) | |  |
| PDHA2 | Testis | | \ | Testis | No expression data | |  |
| **PHF7** | Testis | | Testis | Testis | Several tissues with strong, Testis (Moderate) | |  |
| PIWIL1 | Testis | | Testis | Testis | Several tissues with strong, Testis (Moderate) | |  |
| POLR2D | Testis | | - | Testis | Several tissues with strong, Testis (Moderate) | |  |
| **PPM1G** | Testis | | - | Testis | Many tissues with strong, Testis (Strong) | |  |
| **PRM1** | Testis | | Testis | Testis | Muscle (Strong), Testis (Moderate) | |  |
| **PRM2** | Testis | | Testis | Testis | No Strong, Testis (Moderate) | |  |
| PRND | Testis | | Testis | Testis | Kidney (Strong), Testis (Moderate) | |  |
| ROPN1 | Testis | | Testis | Testis | Many tissues with strong, Testis (Strong) | |  |
| **RPL39L** | Testis | | - | Testis | Many tissues with strong, Testis (Strong) | |  |
| RUVBL2 | Testis | | - | Testis | Many tissues with strong, Testis (Strong) | |  |
| SLC25A31 | Testis | | Testis | Testis | Several tissues with strong, Testis (Moderate) | |  |
| SLC6A16 | Testis | | Testis | Testis | No Strong | |  |
| **SPANXC** | Testis | | \ | Testis | Several tissues with strong, Testis (Moderate) | |  |
| SPATA6 | Testis | | - | Testis | Several tissues with strong, Testis (Strong) | |  |
| **SURF2** | Testis | | - | Testis | Several tissues with strong, Testis (Moderate) | |  |
| TAF7L | Testis | | Testis | Testis | Testis (Strong) | |  |
| TBL2 | Testis | | - | Testis | Several tissues with strong, Testis (Strong) | |  |
| **TBPL1** | Testis | | Bone marrow | Testis | No expression data | |  |
| **TCP11** | Testis | | Testis | Testis | No strong, Testis (Moderate) | |  |
| **TEX14** | Testis | | Testis | Testis | Many tissues with strong, Testis (Moderate) | |  |
| **TNP1** | Testis | | Testis | Testis | No Strong, Testis (Moderate) | |  |
| TPTE | Testis | | Testis | Testis | No expression data | |  |
| **TULP2** | Testis | | \ | Testis | No expression data | |  |
| **PRSS16** | Thymus | | - | Thymus | Pancreas (Strong), Stomach (Strong) | |  |
| CD1A | Thymus | | \ | Thymus | Skin (Strong) | |  |
| CD1B | Thymus | | \ | Thymus | Many tissues with strong | |  |
| **CD1E** | Thymus | | Thymus | Thymus | No expression data | |  |
| FBXL12 | Thymus | | - | - | No expression data | |  |
| NFATC3 | Thymus | | - | Thymus | Many tissues with strong | |  |
| RAG1 | Thymus | | Thymus, Tesitis | Thymus | No expression data | |  |
| **TPO** | Thyroid | | - | Thyroid, Spleen | Thyroid (Strong) | |  |
| **TG** | Thyroid | | Larynx, Tongue | Testis, Kidney, Spleen | Thyroid (Strong) | |  |
| SLC26A4 | Thyroid | | - | Thyroid | Several tissues with strong, Thyroid (Moderate) | |  |
