# Supplementary material for: Combining Evidence of Preferential Gene-Tissue Relationships from Multiple Sources
Source: PLoS One. 2013 Aug 12;8(8):e70568. doi: 10.1371/journal.pone.0070568 (PMC3741196; doi:10.1371/journal.pone.0070568)
Supplement: Table S8 — Comparison of 2-Selective Genes. (DOCX) [file pone.0070568.s010.docx]

Table S8 Comparison of 2-Selective Genes.

Comparing the result of and 31 2-selective genes with medium score in the best coverage with TiGER, PaGenBase and HPA. Gray shows the exact agreement in the corresponding database, red shows partially agreement, and absence and disagreement remain white. ‘\’ means the gene is not found in the database and ‘-‘ means this gene is not specific.

|  | **PREDICTED** | **TIGER** | **PAGENBASE** | **HPA** |
| --- | --- | --- | --- | --- |
| **ATP4A** | Adrenal, Stomach | \ | Adrenal, Stomach, Cerebellum | Stomach (Strong), Adrenal (Moderate) |
| **GABRD** | Cerebellum, CNS | CNS | Cerebellum, CNS | No expression data |
| **PMP2** | Spinal cord, CNS | CNS | CNS | No expression data |
| **GFAP** | Spinal cord, CNS | CNS | Spinal cord, CNS | No strong, CNS (Moderate) |
| **CKMT2** | Heart, Muscle | Heart, Muscle, Kidney | Heart | Heart (Strong), Stomach (Strong) |
| **FBXO40** | Heart, Muscle | - | Heart, Muscle | Several tissues with strong, Heart (Moderate) |
| **SYNPO2L** | Heart, Muscle | Heart, Muscle | Heart | No expression data |
| **MYL2** | Heart, Muscle | Heart, Muscle | Heart, Muscle | Several tissues with strong, Heart (Strong), Muscle (Strong) |
| **MYH7** | Heart, Muscle | Heart, Muscle | Muscle | Heart, Muscle |
| **MYOM2** | Heart, Muscle | Heart, Muscle | Heart | Heart, Muscle |
| **CYP3A4** | Liver, Small intestine | Liver | Liver | Liver, Small intestine, Duodenum |
| **IGFBP1** | Liver, Placenta | Placenta | Placenta | Placenta (Strong), Liver (Weak) |
| **HAO2** | Kidney, Liver | Kidney, Liver | Kidney, Liver | No expression data |
| **OTC** | Kidney, Liver | \ | Small intestine | Liver, Stomach, Duodenum |
| **KHK** | Kidney, Liver | Liver | Liver | Kidney, Liver, Duodenum |
| **GLYAT** | Kidney, Liver | Kidney, Liver | Kidney, Liver | No expression data |
| **SULT2A1** | Adrenal, Liver | Liver | Liver, Adrenal | Several tissues with strong, Liver (Strong), Adrenal (Strong) |
| **CLDN18** | Lung, Stomach | Stomach | Lung | Stomach (Strong) |
| **FCN3** | Lung, Liver | - | Liver | Adrenal (Strong), Lung (Moderate), Liver (Weak) |
| **PCSK1** | Pancreas, CNS | Pancreas | Pancreas | Several tissues with strong, CNS (Strong) |
| **HSD3B1** | Placenta, Adrenal | Placenta | Placenta | No expression data |
| **CGA** | Placenta, CNS | Placenta | Placenta, Pituitary tissue | Placenta |
| **RAB3B** | Placenta, Prostate | - | Placenta, Prostate, CNS | Placenta (Moderate), Prostate (Strong) |
| **PDE6G** | Retina, Pancreas | Eye | Retina, Spleen | No expression data |
| **SIX3** | Retina, CNS | \ | Retina, CNS | No expression data |
| **MLANA** | Skin, Retina | Skin | Skin, Retina | Skin (Strong) |
| **LIPF** | Small intestine, Stomach | Stomach | Stomach, Kidney, Small intestine | Stomach |
| **CHD5** | Testis, CNS | CNS | Testis, CNS | Several tissues with Strong, testis (Moderate), CNS (Moderate) |
| **CCL25** | Thymus, Small intestine | Thymus | Thymus | Several tissues with Strong, Small intestine (Moderate) |
| **PLUNC** | Tongue, Trachea | Larynx, Lung | Trachea | No expression data |
| **MSMB** | Prostate, Trachea | Prostate | Prostate, Lung | Prostate, Stomach, Bronchus |
