# Supplementary material for: Combining Evidence of Preferential Gene-Tissue Relationships from Multiple Sources
Source: PLoS One. 2013 Aug 12;8(8):e70568. doi: 10.1371/journal.pone.0070568 (PMC3741196; doi:10.1371/journal.pone.0070568)
Supplement: Table S9 — The frequency of detected tissues. (DOCX) [file pone.0070568.s011.docx]

**Table S9 -** The frequency of detected tissues among the 1462 specific genes.

| **Tissue name** | **Frequency** | **Relative frequency** |
| --- | --- | --- |
| Testis | 669 | 0.45759 |
| Liver | 123 | 0.08413 |
| Placenta | 96 | 0.06566 |
| CNS | 77 | 0.05267 |
| Muscle | 75 | 0.05130 |
| Pancreas | 47 | 0.03215 |
| Kidney | 44 | 0.03010 |
| SalivaryGland | 41 | 0.02804 |
| Skin | 26 | 0.01778 |
| Heart | 24 | 0.01642 |
| Thymus | 22 | 0.01505 |
| Blood | 19 | 0.01300 |
| SmallIntestine | 17 | 0.01163 |
| Cerebellum | 16 | 0.01094 |
| Thyroid | 15 | 0.01026 |
| Lung | 15 | 0.01026 |
| Adrenal | 14 | 0.00958 |
| Trachea | 12 | 0.00821 |
| Tonsil | 10 | 0.00684 |
| Retina | 10 | 0.00684 |
| BoneMarrow | 10 | 0.00684 |
| Bonemarrow | 9 | 0.00616 |
| Prostate | 9 | 0.00616 |
| Bile | 7 | 0.00479 |
| SpinalCord | 7 | 0.00479 |
| Ovary | 7 | 0.00479 |
| Epididymis | 6 | 0.00410 |
| leukocyte | 6 | 0.00410 |
| MeniscusJoint | 4 | 0.00274 |
| Vessel | 4 | 0.00274 |
| Esophagus | 3 | 0.00205 |
| Colon | 2 | 0.00137 |
| Spleen | 2 | 0.00137 |
| Breast | 2 | 0.00137 |
| Uterus | 2 | 0.00137 |
| Stomach | 2 | 0.00137 |
| MammaryGland | 1 | 0.00068 |
| Tongue | 1 | 0.00068 |
| Endometrium | 1 | 0.00068 |
| Vulva | 1 | 0.00068 |
| VasDeferens | 1 | 0.00068 |
| FallopianTube | 1 | 0.00068 |
| BoneStructure | 1 | 0.00068 |
| ArticularSurfaceofBone | 1 | 0.00068 |
