# Supplementary material for: Combining Evidence of Preferential Gene-Tissue Relationships from Multiple Sources
Source: PLoS One. 2013 Aug 12;8(8):e70568. doi: 10.1371/journal.pone.0070568 (PMC3741196; doi:10.1371/journal.pone.0070568)
Supplement: Table S10 — The frequency of pairs of detected tissues. (DOCX) [file pone.0070568.s012.docx]

**Table S10 -** The frequency of pairs of detected tissues among the 214 2-selective genes with strong support. Only those with frequencies above five are shown.

| Tissue pairs | Frequency | Relative Frequency |
| --- | --- | --- |
| WholeBlood&leukocyte | 37 | 0.087 |
| CNS&SpinalCord | 35 | 0.083 |
| Heart&Muscle | 26 | 0.061 |
| Kidney&Liver | 12 | 0.028 |
| Pancreas&SmallIntestine | 11 | 0.026 |
| CNS&Placenta | 10 | 0.024 |
| Placenta&Testis | 9 | 0.021 |
| CNS&Cerebellum | 9 | 0.021 |
| Pancreas&Testis | 9 | 0.021 |
| Epididymis&Testis | 8 | 0.019 |
| Lymph&Testis | 8 | 0.019 |
| CNS&Testis | 8 | 0.019 |
| Liver&SmallIntestine | 7 | 0.017 |
| Muscle&Testis | 7 | 0.017 |
| Muscle&SmallIntestine | 7 | 0.017 |
| CNS&Pineal | 7 | 0.017 |
| Testis&Trachea | 5 | 0.012 |
| Lymph&Thymus | 5 | 0.012 |
