# Supplementary material for: Combining Evidence of Preferential Gene-Tissue Relationships from Multiple Sources
Source: PLoS One. 2013 Aug 12;8(8):e70568. doi: 10.1371/journal.pone.0070568 (PMC3741196; doi:10.1371/journal.pone.0070568)
Supplement: Table S11 — Comparision of the predicted results of the 13 drug target genes with TiGER, PaGenBase and HPA. (DOCX) [file pone.0070568.s013.docx]

**Table S11** Comparing the predicted results of the 13 drug target genes with TiGER, PaGenBase and HPA. A gray color indicates exact agreement and a red color a partial agreement. The predicted results are when using all 4 datasets.

| TARGET GENES | Predicted | TiGER | PaGenBase | HPA |
| --- | --- | --- | --- | --- |
| ATP4A | Stomach, Adrenal | \ | Stomach, Cerellum, Adrenal | Stomach |
| SCN5A | Heart | - | Heart | No expression data |
| PNLIP | Pancreas | Pancreas | Pancreas, Adipose | No expression data |
| LIPF | Stomach, Small intestine | Stomach | Small intestine, Kidney, Stomach | Stomach |
| TPO | Thyroid | - | Thyroid, Spleen | Thyroid |
| SLC5A2 | Kidney | Kidney, Testis | Kidney | Kidney, and several others |
| CRP | Liver | Liver | Liver | Liver |
| KLK3 | Prostate | Mammary gland, Prostate | Prostate, Colon | Prostate |
| TNNT2 | Heart | Heart | Heart | Heart |
| TG | Thyroid | Larynx, Tongue | Thyroid | Thyroid |
| SLC26A4 | Thyroid | - | Thyroid, Kidney | - |
| IYD | Thyroid | Kidney | Thyroid | No expression data |
| TSHR | Thyroid | Thyroid | Thyroid, Breast, Liver | Thyroid |
