# Supplementary material for: Combining Evidence of Preferential Gene-Tissue Relationships from Multiple Sources
Source: PLoS One. 2013 Aug 12;8(8):e70568. doi: 10.1371/journal.pone.0070568 (PMC3741196; doi:10.1371/journal.pone.0070568)
Supplement: Table S13 — Shows the predicted tissue for each of the datasets for the 13 drug target genes. (DOCX) [file pone.0070568.s015.docx]

**Table S13** Shows the predicted tissue for each of the datasets for the 13 drug target genes.

|  | **GNF1H** | **GeAZr** | **GSE3113** | **GSE7307** | **Target** |
| --- | --- | --- | --- | --- | --- |
| **ATP4A** | Adrenal | Stomach | Adrenal | Stomach | **Stomach** |
| **SCN5A** | Heart | Heart | Heart |  | **Heart** |
| **PNLIP** | Pancreas | Pancreas, Bile | Pancreas | Pancreas, Small intestine | **Pancreas** |
| **LIPF** | Small intestine | Stomach | Spinal cord, Small intestine | Stomach, Small intestine | **Stomach** |
| **TPO** | Thyroid | Thyroid | Thyroid | Thyroid | **Thyroid** |
| **SLC5A2** | Kidney | Kidney | Kidney | Kidney | **Kidney** |
| **CRP** | Pancreas | Liver | Liver | Liver, Small intestine | **Liver** |
| **KLK3** | Prostate | Prostate | Prostate | Prostate, Urethra | **Prostate** |
| **TNNT2** | Heart | Heart | Heart | Heart | **Heart** |
| **TG** | Thyroid | Thyroid | Thyroid | Thyroid | **Thyroid** |
| **SLC26A4** | Thyroid | Thyroid | Thyroid | Thyroid | **Thyroid** |
| **IYD** | \ | Thyroid | \ | \ | **Thyroid** |
| **TSHR** | Thyroid | Thyroid | Thyroid | Thymus, Thyroid | **Thyroid** |
