# Supplementary material for: Combining Evidence of Preferential Gene-Tissue Relationships from Multiple Sources
Source: PLoS One. 2013 Aug 12;8(8):e70568. doi: 10.1371/journal.pone.0070568 (PMC3741196; doi:10.1371/journal.pone.0070568)
Supplement: Text S1 — Additional information around method, data and results. (DOCX) [file pone.0070568.s016.docx]

Supplementary material:

Combining evidence of preferential Gene-Tissue relationships from multiple sources

**Authors:** Jing Guo^1^, Mårten Hammar^2^, Lisa Öberg^3^, Shanmukha S. Padmanabhuni^4^, Marcus Bjäreland^5^ and Daniel Dalevi^6*^

^1^ Department of Medical Biochemistry and Biophysics, Karolinska Institute, S-17177, Stockholm, Sweden

^2^Cardiovascular & Gastrointestinal iMed, AstraZeneca R&D Mölndal, S-43183 Mölndal, Sweden

^3^Respiratory, Inflammation & Autoimmune iMed, AstraZeneca R&D Mölndal, S-43183 Mölndal, Sweden

^4^DERI, IDA Business park, Galway, Ireland

^5^R&D Information, AstraZeneca R&D Mölndal, S-43183 Mölndal, Sweden

^6^Biometrics and Information Sciences, AstraZeneca R&D Mölndal, S-43183 Mölndal, Sweden

* To whom correspondence should be addressed.

# Methods

## ROKU-SPM

*SPM*

The SPM proposed by the PaGenBase group is described as “the ratio of vector$X_{i}$’s scalar projection in the direction of vector $X_{p}$ against the length of $X_{p}$”. As the projection can be calculated in many manners (absolute value, squared value, etc.), we use a squared projection in our method, which results in this formula:

${SPM}_{g}=\frac{x_{t}^{2}}{\sum_{t=1}^{N} x_{t}^{2}}$ ,

where *N* is the total number of tissues, $g$ stands for a gene, and $x_{t}$ is the expression intensity of a gene in tissue *t*.

*ROKU*

According to the original paper (Kadota, Ye et al. 2006), Tukey’s biweight, $T_{bw}$, is used to improve the robustness before Shannon entropy is applied:

$x_{t}^{'}=\left| x_{t}-T_{bw} \right|$,

where $x_{t}$ is the expression intensity of a gene in tissue *t*.

The Shannon entropy is calculated as

$H\left( x \right)=-\sum_{1}^{N} p_{t}\text{log}_{2}p_{t}$,

where $p_{t}$ is the relative expression of $x_{t}$ for tissue t defined as

$p_{t}= \frac{x_{t}}{\sum_{t=1}^{N} x_{t}}$ ,

A simplified AIC method is used to detect the outliers, which in our case, are the specific tissues.

*ROKU-SPM*

Although there are good examples, the actual results of ROKU and SPM were not performing sufficiently on most of the training data compared to the other methods. In general, there are two problems:

- For the ROKU method, there are cases where the entropies are incredibly low while a large number of outliers are detected.
- When the data is noisy (GDS raw data), the difference between the entropy of specific and non-specific genes is hardly detectable. Similarly, for the SPM method, the *SPM* value of the specific tissue is not remarkable different to the other non-specific tissues.

For example, to illustrate the problems, we look at the probe set *214421_x_at* for gene CYP2C9. The figure below shows the expression distribution in GNF1H (“BioGPS”, left) and GDS596 (right). In GNF1H, although low entropy (0.527) and high SPM (0.99) supporting specificity for Liver, which is also easily caught by eye-browsing, the outlier detection method gives us 6 specific tissues (i.e. Problem 1). In GDS596, on the other hand, we have high entropy (5.75) and low SPM (0.02) for Liver, this gene can hardly be identified as specific based on either the Entropy or SPM. The outlier detection method, however, correctly identifies Liver as a specific tissue (i.e. Problem 2).


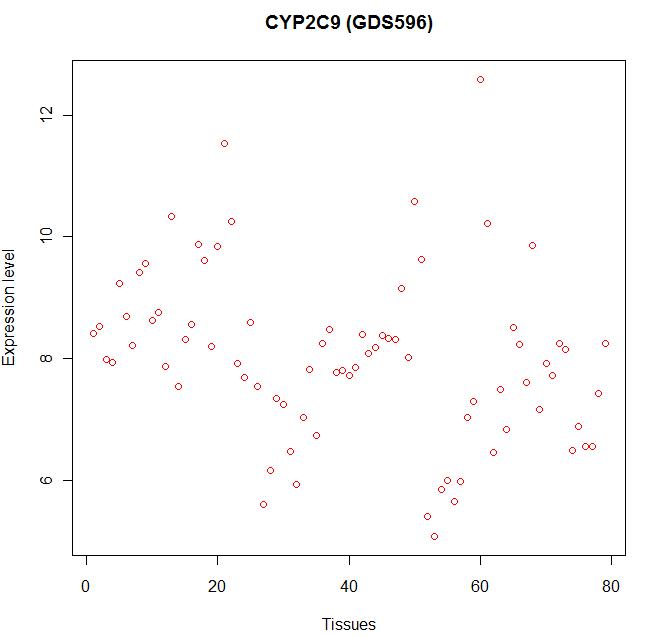

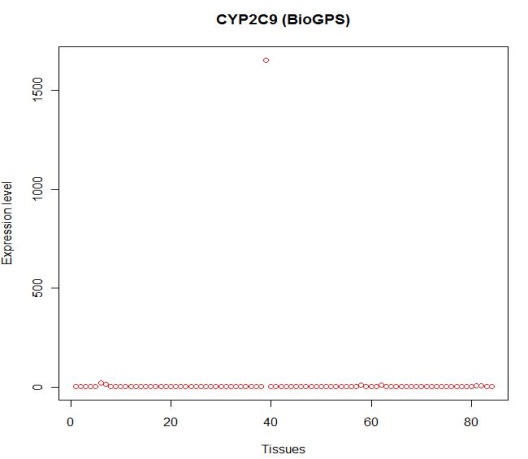


We propose an improved method, which combines ROKU and SPM, to resolve the two issues, which we will refer to as ROKU-SPM. In the ROKU-SPM method, the *SPM* value is introduced as a parameter to the ROKU method. A specifically expressed gene must satisfy the following requirements:

- The entropy is lower than $E$ - the Entropy threshold.
- The outlier with the largest value is greater than ${SPM}_{1}$ – the first SPM threshold.


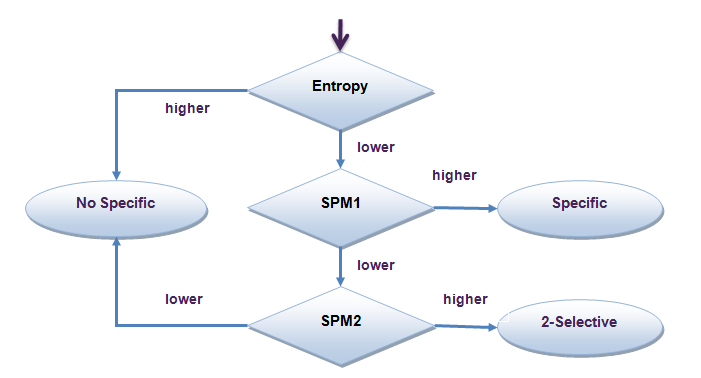
Similarly, the requirements for 2-selective genes:

- The entropy is lower than $E$.
- The outlier with the 2^nd^ largest value is greater than ${SPM}_{2}$ – the second SPM threshold.

The flow of the ROKU-SPM procedure

## Decision function

This method gives a deterministic parameter ($d$) for gene specificity based on *gap* and a significance probability ($sp$). The $gap$ indicates the absolute difference between the intensities of two tissues; the significance probability is calculated by a Dixon test:

$$sp=P\left[ t\geq T_{critical} \right]=1-\int_{0}^{T_{critical}} F_{2,2n-2}\left( z \right)dz ,$$

where $T_{critical}$ is the Dixon critical statistic, $n$ is the total number of tissues, $F$ is the standard statistical $F$ distribution with $(2,2n-2)$ degrees of freedom.

The indicator of gene specificity is calculated by a decision function:

$$d\left( g,s \right)=1-\left[ \left( 1-s \right)^{\alpha}\left( 1-g \right)^{\beta}\left( \frac{\delta\left( 1-g \right)+\left( 1-\delta\right)\left( 1-s \right)}{\left( 1-g \right)+\left( 1-s \right)} \right)^{\gamma} \right]^{\phi} ,$$

where $s$ and $g$ are the variant of the *gap* and *sp* parameters (see the original paper). $\alpha=\beta=\gamma=1.5$ and $\delta={(\alpha+\beta+\gamma)}^{-1}=0.3$ are independent parameters chosen empirically by the authors of the original paper.

## Bayes factor

See original paper for description. The procedure for testing $H_{1}$ and $H_{1}^{(2)}$ are:

1. Test $H_{1}^{(2)}$ if supported, output result as 2-selective and STOP.
2. Test $H_{1}$ if supported, output result as specific, STOP.
3. Output result as ubiquitous.

## Optimization function

See original paper.

## Training and test gene sets

The data for the training set are chosen from the supplemental information of HugeIndex.org (http://zlab.bu.edu/HugeIndex/PaperInfo/Supplement_3-tissue-selective-genes.html), under the group of ‘brain’, ‘kidney’, ‘liver’, ‘lung’, ‘muscle’, ‘prostate’ and ‘vulva’ specific. The parameter training is based on a combination of all specific gene sets and 10 ubiquitous expressed genes chosen from the “Housekeeping” gene sets. To assess the training result, parameters are also trained on 4 other gene sets, each of which contain 10 specific genes and 10 ubiquitous expressed genes. The 4 assessment training sets are listed below.

|  | Kidney Set | Muscle Set | Lung, Prostate Set | Liver |
| --- | --- | --- | --- | --- |
| Tissue Specific Genes | AQP2 | MYOM2 | HOXB13 | FT2 |
|  | PEPD | MYOM1 | FCN3 | CYP2CT8 |
|  | SLC34A1 | MYBPC2 | SEMG1 | CYP2C9 |
|  | UMOD | FBP2 | ARG2 | KLKB1 |
|  | FMO1 | SLN | MARCO | C8G |
|  | SLC5A2 | UCP3 | CLDN18 | CYP3A7 |
|  | SLC12A1 | MYL1 | NPY | TDO2 |
|  | KCNJ1 | TNNC2 | DUSP1 | CRP |
|  | SLC12A3 | ACTN2 | PGC | MBL2 |
|  | CLCNKB | RPL3L | LAMP3 | SERPINCT |
| Ubiquitously Expressed  Genes | NACA | SURF1 | RPL19 | RPL29 |
|  | RPL11 | JUNB | CD63 | H3F3B |
|  | QARS | COX7C | WARS | RPS26 |
|  | SSR2 | RPL31 | UBA52 | BAT |
|  | RPL3 | HSPB1 | HLA-E | SURFT |
|  | RPL6 | EEF1D | RPL23 | RPL8 |
|  | RPS18 | RPL41 | RPL17 | RPL38 |
|  | SERPINA3 | CFL1 | FLNA | COMT |
|  | PRDX1 | SARS | RPL35A | RPS7 |
|  | RPL13 | CTNNB1 | EEF2 | HSPBT |

## Training Schema

The purpose of the optimization process is to find the best parameters of each method on each dataset. All sets of training genes are used in this process. The agreement between the actual result and the expected result is measured by the optimization function.

The procedure of training:

- Constrain each parameter to an interval according to the distribution of the parameter itself. For example, the entropy of GNF1H data is between 0.045 and 6.110 (the first quantile, 25%, is 4.444). As we assume that the proportion of specific genes among all genes is no larger than 25%, we use the range from 0.045 to 4.444 as our preset scope to optimize. The same principle is applied to other parameters.
- Run loops to estimate the combination of parameters. This step is repeated several times beginning with large steps on the whole interval to find approximate values. Then we use smaller steps to fine prune the parameters over specific intervals around those approximate values.

The parameters after training on the Mix gene set:

Parameter set 1): Mix

|  | ROKU-SPM | | | Decision Function | | $\boldsymbol{BF}$ | | | |
| --- | --- | --- | --- | --- | --- | --- | --- | --- | --- |
|  | $\boldsymbol{E}$ | $\boldsymbol{SPM}_{\mathbf{1}}$ | $\boldsymbol{SPM}_{\mathbf{2}}$ | $\boldsymbol{min}(\boldsymbol{s})$ | $\boldsymbol{max}(\boldsymbol{s})$ | $\boldsymbol{BF}_{\boldsymbol{1}}$ | $c1$ | $\boldsymbol{BF}_{\boldsymbol{2}}$ | $c2$ |
|  |  |  |  |  |  |  |  |  |  |
| GNF1H | 3.5 | 0.65 | 0.4 | -4 | -12 | 46 | 1.7 | 1155 | 1.79 |
| GeAZr | 4.93 | 0.41 | 0.25 | -5 | -15 | - |  | - | - |
| GDS3113 | 4.35 | 0.05 | 0.055 | -1 | -3 | 32 | 1.79 | 1200 | 1.92 |
| GSE7307 | 4.9 | 0.035 | 0.04 | -3 | -5 | 38 | 1.79 | 2007 | 1.64 |

Note: The threshold $E$ (Entropy), ${SPM}_{1}$ and ${SPM}_{2}$ are optimized for ROKU-SPM. $s$*,* $g$and $d$ are optimized for the decision function method. ${BF}_{1}, {BF}_{2}$and $c$ are optimized for the Bayes factor method. Same annotation is used below for other training sets.

Similarly, the parameters trained on other sets are listed below:

Parameter set 2): kidney-specific

|  | ROKU-SPM | | | Decision Function | | $\boldsymbol{BF}$ | | | |
| --- | --- | --- | --- | --- | --- | --- | --- | --- | --- |
|  | $\boldsymbol{E}$ | $\boldsymbol{SPM}_{\mathbf{1}}$ | $\boldsymbol{SPM}_{\mathbf{2}}$ | $\boldsymbol{min}(\boldsymbol{s})$ | $\boldsymbol{max}(\boldsymbol{s})$ | $\boldsymbol{BF}_{\boldsymbol{1}}$ | $c1$ | $\boldsymbol{BF}_{\boldsymbol{2}}$ | $c2$ |
|  |  |  |  |  |  |  |  |  |  |
| GNF1H | 3.80 | 0.45 | 0.30 | -5 | -13 | 46 | 1.7 | 1155 | 1.79 |
| GeAZr | 4.84 | 0.36 | 0.20 | -4 | -13 | - |  | - | - |
| GDS3113 | 4.23 | 0.055 | 0.045 | -1 | -4 | 32 | 1.79 | 1200 | 1.92 |
| GSE7307 | 4.80 | 0.03 | 0.03 | -3 | -5 | 38 | 1.79 | 2007 | 1.64 |

Parameter set 3): muscle-specific

|  | ROKU-SPM | | | Decision Function | | $\boldsymbol{BF}$ | | | |
| --- | --- | --- | --- | --- | --- | --- | --- | --- | --- |
|  | $\boldsymbol{E}$ | $\boldsymbol{SPM}_{\mathbf{1}}$ | $\boldsymbol{SPM}_{\mathbf{2}}$ | $\boldsymbol{min}(\boldsymbol{s})$ | $\boldsymbol{max}(\boldsymbol{s})$ | $\boldsymbol{BF}_{\boldsymbol{1}}$ | $c1$ | $\boldsymbol{BF}_{\boldsymbol{2}}$ | $c2$ |
|  |  |  |  |  |  |  |  |  |  |
| GNF1H | 3.00 | 0.60 | 0.40 | -3 | -9 | 46 | 1.7 | 1155 | 1.79 |
| GeAZr | 4.63 | 0.45 | 0.31 | -3 | -9 | - |  | - | - |
| GDS3113 | 4.33 | 0.065 | 0.05 | -1 | -3 | 32 | 1.79 | 1200 | 1.92 |
| GSE7307 | 4.95 | 0.05 | 0.04 | -5 | -8 | 38 | 1.79 | 2007 | 1.64 |

Parameter set 4): lung-specific and prostate-specific

|  | ROKU-SPM | | | Decision Function | | $\boldsymbol{BF}$ | | | |
| --- | --- | --- | --- | --- | --- | --- | --- | --- | --- |
|  | $\boldsymbol{E}$ | $\boldsymbol{SPM}_{\mathbf{1}}$ | $\boldsymbol{SPM}_{\mathbf{2}}$ | $\boldsymbol{min}(\boldsymbol{s})$ | $\boldsymbol{max}(\boldsymbol{s})$ | $\boldsymbol{BF}_{\boldsymbol{1}}$ | $c1$ | $\boldsymbol{BF}_{\boldsymbol{2}}$ | $c2$ |
|  |  |  |  |  |  |  |  |  |  |
| GNF1H | 3.85 | 0.50 | 0.35 | -5 | -13 | 46 | 1.7 | 1155 | 1.79 |
| GeAZr | 4.81 | 0.44 | 0.38 | -3 | -8 | - |  | - | - |
| GDS3113 | 4.44 | 0.05 | 0.03 | -1 | -3 | 32 | 1.79 | 1200 | 1.92 |
| GSE7307 | 4.54 | 0.04 | 0.03 | -3 | -4 | 38 | 1.79 | 2007 | 1.64 |

Parameter set 5): liver-specific

|  | ROKU-SPM | | | Decision Function | | $\boldsymbol{BF}$ | | | |
| --- | --- | --- | --- | --- | --- | --- | --- | --- | --- |
|  | $\boldsymbol{E}$ | $\boldsymbol{SPM}_{\mathbf{1}}$ | $\boldsymbol{SPM}_{\mathbf{2}}$ | $\boldsymbol{min}(\boldsymbol{s})$ | $\boldsymbol{max}(\boldsymbol{s})$ | $\boldsymbol{BF}_{\boldsymbol{1}}$ | $c1$ | $\boldsymbol{BF}_{\boldsymbol{2}}$ | $c2$ |
|  |  |  |  |  |  |  |  |  |  |
| GNF1H | 3.80 | 0.58 | 0.32 | -5 | -10 | 46 | 1.7 | 1155 | 1.79 |
| GeAZr | 4.75 | 0.42 | 0.33 | -3 | -8 | - |  | - | - |
| GDS3113 | 4.23 | 0.045 | 0.025 | -1 | -3 | 32 | 1.79 | 1200 | 1.92 |
| GSE7307 | 4.67 | 0.04 | 0.035 | -3 | -6 | 38 | 1.79 | 2007 | 1.64 |

## Vocabulary mapping

The list of tissues before and after grouping is shown in Table S1. To avoid the bias of using only one certain tissue to represent the grouped tissue, we selected the tissue with the highest expression value within the group to be the representative (for the datasets with replicates, tissue with the highest average of sample expression value are used).

# Results

## Training and optimization

We clustered the results from each dataset using a measure of similarity based on the results of each method (see Fig.1). We also added the databases (PaGenBase, TiGER and HPA). A standard hierarchical clustering was done with distance value ($d_{k}\left( i,j \right)$) defined by a simple similarity measure. For each gene, if the result is the same, the distance between them will be 0; if partially same, then 0.5 (that is one out of two tissues agree), and if not the same, the distance will be 1.

$$d_{k}\left( i,j \right)= \left\{ \begin{aligned} \text{0, if the result of method i and j are the same } \\ \text{0.5, if the result of method i and j are partially the same} \\ \text{1, }\text{if the result of method i and j are different } \end{aligned} \right.$$

Where *k* is the number of gene, and *i, j* are the number of methods. The total distance between two methods is the sum of distances of the training set ($N=30$).

$D\left( i,j \right)= \sum_{k=1}^{N} d_{k}(i,j)$ .

The distance matrix is formed by $D\left( i,j \right)$ and the R-package hclust is used to perform the clustering.

## Results from combined output of all genes

As shown in Table 5 in the manuscript, there are 191 genes detected as specific with *strong support*, $\boldsymbol{t}_{\boldsymbol{s}}(\boldsymbol{T})=\boldsymbol{1}$, and 31 2-selective genes with support from all five datasets, 4 out of 4 (31 with *medium-high* support $\left( \boldsymbol{t}_{\boldsymbol{s}}\left( \boldsymbol{T}_{\boldsymbol{1}} \right),\boldsymbol{t}_{\boldsymbol{s}}\left( \boldsymbol{T}_{\boldsymbol{2}} \right) \right)\geq(\boldsymbol{0}.\boldsymbol{3},\boldsymbol{0}.\boldsymbol{3})$). These results are supported by all methods and should be detectable by any of the data source individually and constitutes the most reliable we have. Therefore we compare them with the results from PaGenBase, TiGER and HPA. Table S7 shows the comparison of the 191 specific genes and a concise version is shown in Table 6 in the manuscript. Table S8 shows the comparison of 31 2-selective genes.

Similar to Figure 5 in the text, the overlap between our predicted results and the results of the databases are shown in Figure S1. Fully agree means that both the resulting tissues must agree. It is expected that the proportions (21% with TiGER, 32% with PaGenBase and 9% with HPA) are much lower than the ones for the specific genes (71% with TiGER, 85% with PaGenBase and 28% with HPA). However, the same numbers for partial agree, i.e. at least one matching tissue, are considerably higher: 61% with TiGER, 68% with PaGenBase and 69% for HPA.

For the 1685 tissue specific genes with strong support in Table 5, we list the frequency of tissues that have been detected as specific in Table S9. Similarly, the 10 most frequently detected tissue pairs among the 346 2-selective genes are shown in Table S10 and Figure S2 (we decided not to list all as most of the tissue pairs only occur once).
